# TDP-43 and other hnRNPs regulate cryptic exon inclusion of a key ALS/FTD risk gene, *UNC13A*

**DOI:** 10.1371/journal.pbio.3002028

**Published:** 2023-03-17

**Authors:** Yuka Koike, Sarah Pickles, Virginia Estades Ayuso, Karen Jansen-West, Yue A. Qi, Ziyi Li, Lillian M. Daughrity, Mei Yue, Yong-Jie Zhang, Casey N. Cook, Dennis W. Dickson, Michael Ward, Leonard Petrucelli, Mercedes Prudencio

**Affiliations:** 1 Department of Neuroscience, Mayo Clinic, Jacksonville, Florida, United States of America; 2 Mayo Clinic Graduate School of Biomedical Sciences, Jacksonville, Florida, United States of America; 3 Center for Alzheimer’s and Related Dementias, National Institute on Aging, NIH, Bethesda, Maryland, United States of America; 4 National Institute of Neurological Disorders and Stroke, NIH, Bethesda, Maryland, United States of America; National Cancer Institute, UNITED STATES

## Abstract

A major function of TAR DNA-binding protein-43 (TDP-43) is to repress the inclusion of cryptic exons during RNA splicing. One of these cryptic exons is in *UNC13A*, a genetic risk factor for amyotrophic lateral sclerosis (ALS) and frontotemporal dementia (FTD). The accumulation of cryptic *UNC13A* in disease is heightened by the presence of a risk haplotype located within the cryptic exon itself. Here, we revealed that TDP-43 extreme N-terminus is important to repress *UNC13A* cryptic exon inclusion. Further, we found hnRNP L, hnRNP A1, and hnRNP A2B1 bind *UNC13A* RNA and repress cryptic exon inclusion, independently of TDP-43. Finally, higher levels of hnRNP L protein associate with lower burden of *UNC13A* cryptic RNA in ALS/FTD brains. Our findings suggest that while TDP-43 is the main repressor of *UNC13A* cryptic exon inclusion, other hnRNPs contribute to its regulation and may potentially function as disease modifiers.

## Abstract 2

This study shows that TDP-43 is the most important repressor of cryptic exon splicing in UNC13A, a risk factor for amyotrophic lateral sclerosis and frontotemporal dementia. While TDP-43 is the main repressor of UNC13A cryptic exon inclusion, other hnRNPs contribute to its regulation and may potentially act as disease modifiers.

## Introduction

Nuclear depletion and cytoplasmic aggregation of TAR DNA-binding protein-43 (TDP-43) is a key pathological feature in more than 97% of amyotrophic lateral sclerosis (ALS) cases and nearly 50% of frontotemporal dementia (FTD) cases (FTLD-TDP) [1–3]. TDP-43 belongs to the heterogeneous nuclear ribonucleoproteins (hnRNPs) family, which largely functions to regulate multiple facets of RNA metabolism, including transcription, alternative splicing, RNA stability, and transport [3–5]. TDP-43 binds to consensus UG repeats within introns or the 3′ UTR of thousands of pre-messenger RNA (mRNA) [4,5]. Further, TDP-43 may interact with other hnRNPs to regulate RNAs [6,7].

Among TDP-43’s various roles, an important function is to repress the inclusion of cryptic exons. Cryptic exons contain parts of introns that are erroneously spliced into the pre-mRNA. Incorporation of cryptic exons may destabilize mRNAs leading to their degradation or alter the reading frame causing the generation of aberrant proteins [8–13]. Similarly, other hnRNPs (C, K, L, M, PTBP1) have also been reported to maintain splicing fidelity by repressing cryptic exon inclusion [6,14–19]. Importantly, hnRNPs (L, A1, A2B1, H1, PTBP1) have been identified as regulators of sortilin splicing, suggesting that multiple protein acting in concert within a network are necessary for splicing of TDP-43 targets [17].

Recently, our group and another group found that the loss of TDP-43 leads to the inclusion of a cryptic exon in *UNC13A* RNA and a reduction in wild-type UNC13A RNA and protein [20,21], which plays a role in neurotransmitter release at the synapse [22–25]. Prior to this finding, genome-wide association studies (GWAS) identified *UNC13A* as top hit for increased risk of ALS and FTD [26–31]. However, the mechanisms underlying this susceptibility remained unknown. *UNC13A* variants associated with ALS/FTD were found within the cryptic exon. Interestingly, FTLD-TDP patients harboring the *UNC13A* risk alleles have increased levels of cryptic exon inclusion and reduced survival time following disease onset [20,21]. Further, the presence of the risk allele (*UNC13A* CE single-nucleotide polymorphism, SNP) enhanced *UNC13A* cryptic exon inclusion by reducing the binding of TDP-43 to *UNC13A* pre-mRNA [21]. Collectively, these studies reveal a connection between genetic risk and TDP-43 function and suggest UNC13A as a candidate for therapeutic intervention. Given the relevance of UNC13A in ALS/FTD pathogenesis, we sought to further clarify the role of TDP-43 in *UNC13A* splicing regulation and explore the contribution of other hnRNPs.

Herein, we provide novel mechanistic insights into *UNC13A* splicing. In addition to the RNA-binding domains, the extreme N-terminal domain, which regulates TDP-43 stability and dimer formation, is also important to repress inclusion of the *UNC13A* cryptic exon. Further, we demonstrated that hnRNP L, hnRNP A1, and hnRNP A2B1 bind *UNC13A* RNA, and their binding is reduced in the presence of the *UNC13A* CE SNP. In FTLD-TDP cases, higher levels of hnRNP L associated with a lower burden of *UNC13A* cryptic RNA accumulation. Further, when TDP-43 protein levels are depleted in human neuronal cells, hnRNP L can reduce *UNC13A* cryptic exon inclusions. Overall, we find that other hnRNPs, particularly hnRNP L, can regulate *UNC13A* splicing in the absence of TDP-43, potentially serving as a disease modifier.

## Results

### The accumulation of *UNC13A* cryptic RNA is sensitive to TDP-43 levels

**Fig 1:**
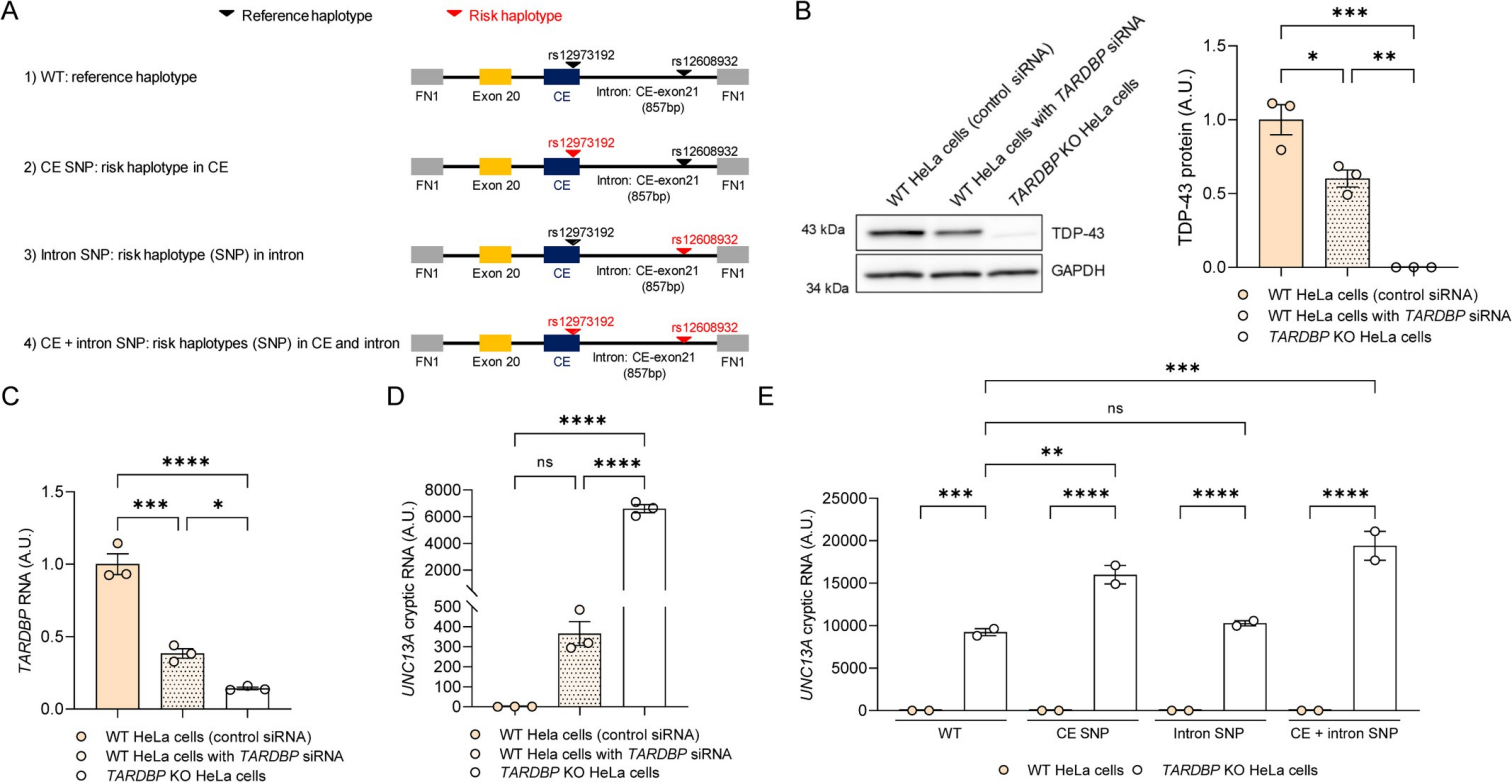
The accumulation of *UNC13A* cryptic RNA is sensitive to TDP-43 levels. (**A**) Schematic representation of the *UNC13A* minigene constructs harboring the GWAS risk variants. The *UNC13A* minigene construct containing the human *UNC13A* cryptic exon sequence and the nucleotide flanking sequences upstream (50 bp at the of end of intron 19, the entire exon 20, the entire intron 20 sequence upstream of the cryptic exon) and downstream (remaining 857 bp intron 20 downstream of the cryptic exon) of the cryptic exon were expressed using the pTB vector. (**B**) Representative immunoblot (left) of cell lysates from each condition using an anti-TDP-43 C-terminal antibody and GAPDH as a loading control. Blots provided in Supporting information (S1 Raw images). Densitometric analysis (right) of TDP-43 protein levels, normalized to GAPDH, confirmed the reduction of TDP-43 in *TARDBP* KO HeLa cells compared to WT HeLa cells expressing either a control or *TARDBP* siRNA. (**C**) qRT-PCR showing *TARDBP* RNA levels in TDP-43 *TARDBP* KO compared to WT HeLa cells expressing a control siRNA or a siRNA against *TARDBP*. (**D**) qRT-PCR shown the enhancement of cryptic exon inclusion in *UNC13A* RNA (WT *UNC13A* minigene) in *TARDBP* KO cells, compared to WT cells treated with *TARDBP* siRNA. (**E**) qRT-PCR of *TARDBP* KO and WT HeLa cells expressing the different *UNC13A* minigene variants (shown in **A**) confirmed the accumulation of *UNC13A* cryptic RNA in *TARDBP* KO cells. Such accumulation was largest in cells containing the cryptic exon SNP (CE SNP and CE + intron SNP). All graphs represent mean ± SEM from 3 (**B–D**) or 2 (**E**) independent experiments. Statistical differences were assessed by one-way ANOVA followed by Tukeys’s (**B–D**) or Bonferroni’s (**E**) multiple comparisons test (ns: not significant, *P < 0.05, **P < 0.005, ***P < 0.0005, ****P < 0.0001). Data used to generate graphs can be found in S3 Table. GWAS, genome-wide association study; qRT-PCR, quantitative reverse transcription polymerase chain reaction; SEM, standard error of mean; siRNA, small interfering RNA; SNP, single-nucleotide polymorphism; TDP-43, TAR DNA-binding protein-43.

To probe factors affecting *UNC13A* splicing, we employed an *UNC13A* minigene splicing assay whereby a construct containing the *UNC13A* cryptic exon, and surrounding sequences (**Fig 1A**), were transiently transfected into HeLa cells and the level of inclusion of the cryptic exon was assessed by quantitative reverse transcription polymerase chain reaction (qRT-PCR).

We first evaluated *UNC13A* cryptic exon splicing using a minigene harboring the reference haplotypes (WT minigene, **Fig 1A**) in wild-type (WT) HeLa cells co-transfected with control small interfering RNA (siRNA) or siRNA targeting *TARDBP*, as well as in TDP-43 knockout (*TARDBP* KO) HeLa cells [32]. TDP-43 protein levels were reduced approximately 50% in si*TARDBP* treated cells and completely depleted in *TARDBP* KO cells compared to WT cells (**Fig 1B**), consistent with a similar decrease in *TARDBP* RNA (**Fig 1C**). We observed a dose-dependent increase in *UNC13A* cryptic exon inclusion based on TDP-43 depletion (**Fig 1D**), revealing that *UNC13A* splicing is highly sensitive to TDP-43 protein levels.

To evaluate how the risk haplotypes may affect this regulation, we generated 3 additional minigene constructs containing the risk haplotype located within the cryptic exon (CE SNP, rs12973192), the SNP located downstream of the cryptic exon (Intron SNP, rs12608932), or both (CE + Intron SNP, rs12973192 and rs12608932) (**Fig 1A**). Expression of all the *UNC13A* minigenes led to a significant accumulation of *UNC13A* cryptic RNA in *TARDBP* KO cells (**Fig 1E**). The accumulation of *UNC13A* cryptic RNA was further enhanced when the risk haplotype was located within the cryptic exon, but not when located within the intron (**Fig 1E**). Of note, expression of high levels of TDP-43_WT,_ but not of a TDP-43 RNA-binding mutant (TDP-43_5FL_: 5 Phe residues; 147, 149, 194, 229, and 231 mutated to Leu in RRM1 and RRM2 [33]), were able to efficiently repress *UNC13A* splicing regardless of risk haplotype (**S1 Fig**).

### The extreme N-terminus of TDP-43 is important for repression of *UNC13A* cryptic exon inclusion

**Fig 2:**
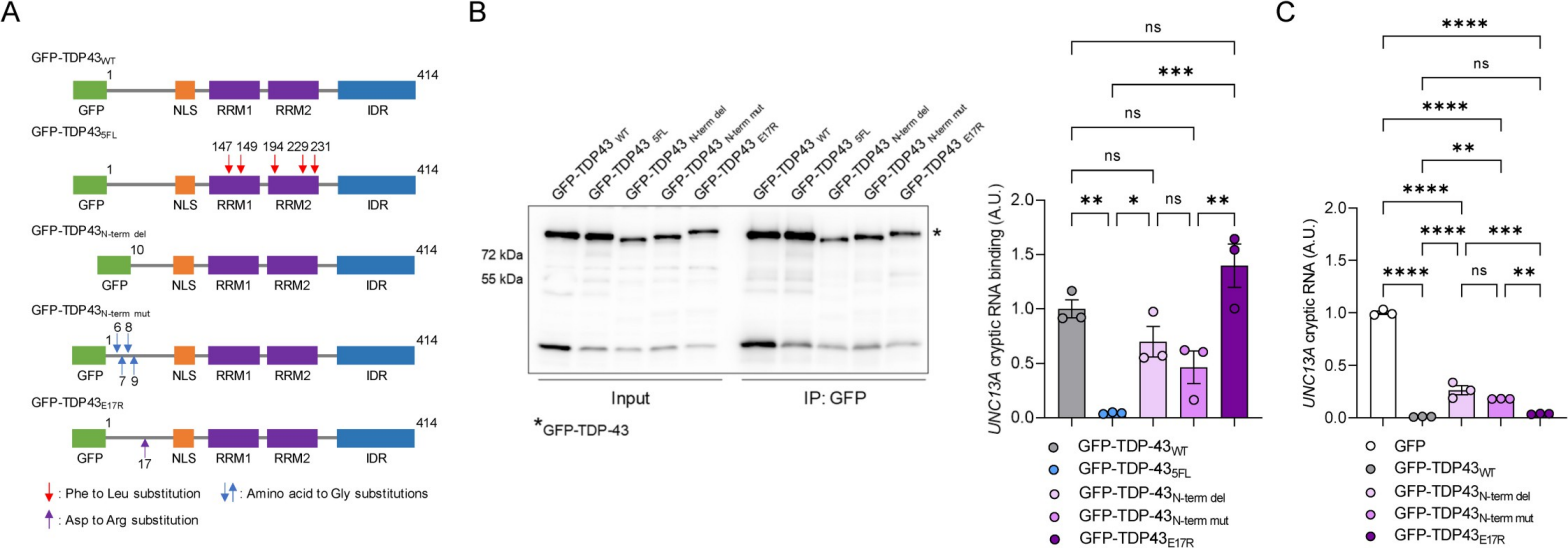
The extreme N-terminus of TDP-43 is required to repress *UNC13A* cryptic exon splicing. (**A**) Schematic representation of GFP-tagged constructs for overexpressing wild-type (GFP-TDP-43_WT_), RNA-binding deficient TDP-43 mutant (GFP-TDP-43_5FL_), N-terminal deletion (GFP-TDP-43_N-term del_), N-terminal mutant (GFP-TDP-43_N-term mut_), and GFP-TDP-43_E17R_ TDP-43. **(B)** *TARDBP* KO HeLa cells were transfected to overexpress the *UNC13A* WT minigene and either GFP-TDP-43_WT_, GFP-TDP-43_5FL_, GFP-TDP-43_N-term del,_ GFP-TDP-43_N-term mut_, or GFP-TDP-43_E17R_. Following transfection, cells were UV-irradiated, and TDP-43 bound RNA was immunoprecipitated using a rabbit polyclonal GFP antibody (Abcam, ab290) as explained in Materials and methods. Representative immunoblots of input and immunoprecipitated samples and from each condition using an anti-GFP antibody (Invitrogen, [C163], 33–2600) (left). Blot provided in Supporting information (S1 Raw images). qRT-PCR (right) analysis shows significantly decreased TDP-43 binding to *UNC13A* RNA only in the cells expressing GFP-TDP-43_5FL_ compared with the cells expressing GFP-TDP-43_WT_. (**C**) qRT-PCR of *UNC13A* cryptic RNA demonstrates the reduced ability of GFP-TDP-43_N-term del,_ GFP-TDP-43_N-term mut_, but not GFP-TDP-43_E17R,_ to rescue *UNC13A* splicing. All graphs represent mean ± SEM of 3 independent replicates. Statistical differences were assessed by one-way ANOVA followed by Tukey’s multiple comparisons test (ns: not significant, *P < 0.05, **P < 0.005, ***P < 0.0005, ****P < 0.0001). Data used to generate graphs can be found in S3 Table. qRT-PCR, quantitative reverse transcription polymerase chain reaction; SEM, standard error of mean; TDP-43, TAR DNA-binding protein-43.

We had previously shown that the extreme N-terminal domain of TDP-43, particularly amino acids 6–9, are critical for stability and dimer formation, as well as splicing of the TDP-43 RNA target, cystic fibrosis transmembrane conductance regulator (*CFTR*) [34]. Further, mutations in amino acid 17 of TDP-43 have also previously shown to disrupt N-terminal domain homotypic interactions [35]. To understand whether dimerization of TDP-43 is required to repress *UNC13A* cryptic exon inclusion, we investigated the ability of TDP-43 N-terminal mutants, GFP-TDP-43_N-term del_ (deletion of amino acids 2–9), GFP-TDP-43_N-term mut_ (R6G, V7G, T8G, E9G) and GFP-TDP-43_E17R_ to bind the *UNC13A* minigene and rescue *UNC13A* splicing, compared to wild-type TDP-43 (GFP-TDP-43_WT_) (**Fig 2A**). The RNA-binding mutant, GFP-TDP-43_5FL_, was used as a negative control (**Fig 2A**). *TARDBP* KO cells were co-transfected with TDP-43 constructs and the *UNC13A* minigene, GFP-tagged TDP-43 constructs were immunoprecipitated and the amount of *UNC13A* RNA bound was assessed by qRT-PCR. All constructs were similarly expressed and efficiently immunoprecipitated (**Fig 2B**, left). Compared to GFP-TDP-43_WT_, perturbations of the RNA-binding domains (GFP-TDP-43_5FL_) showed significantly reduced ability to bind *UNC13A* RNA (**Fig 2B**, right). Interestingly, GFP-TDP-43_N-term del_, GFP-TDP-43_N-term mut_, but not GFP-TDP-43_E17R_, showed reduced binding to *UNC13A* cryptic RNA, although this reduction did not reach statistical significance (**Fig 2B**). Moreover, GFP-TDP-43_N-term del_ and GFP-TDP-43_N-term mut_ showed partial rescue effects of *UNC13A* cryptic splicing compared to control vector (GFP). However, the rescue effects were less than those of GFP-TDP43_WT_ (**Fig 2C**). In contrast, GFP-TDP-43_E17R_ exhibited similar splicing activity to GFP-TDP43_WT_ (**Fig 2C**). Taken together, our results suggest that disruption of extreme TDP-43 N-terminal region impairs TDP-43 ability to fully repress *UNC13A* cryptic exon inclusion through regulation of RNA binding.

### hnRNP L, hnRNP A1, and hnRNP A2B1 bind *UNC13A* RNA independently of TDP-43

**Fig 3:**
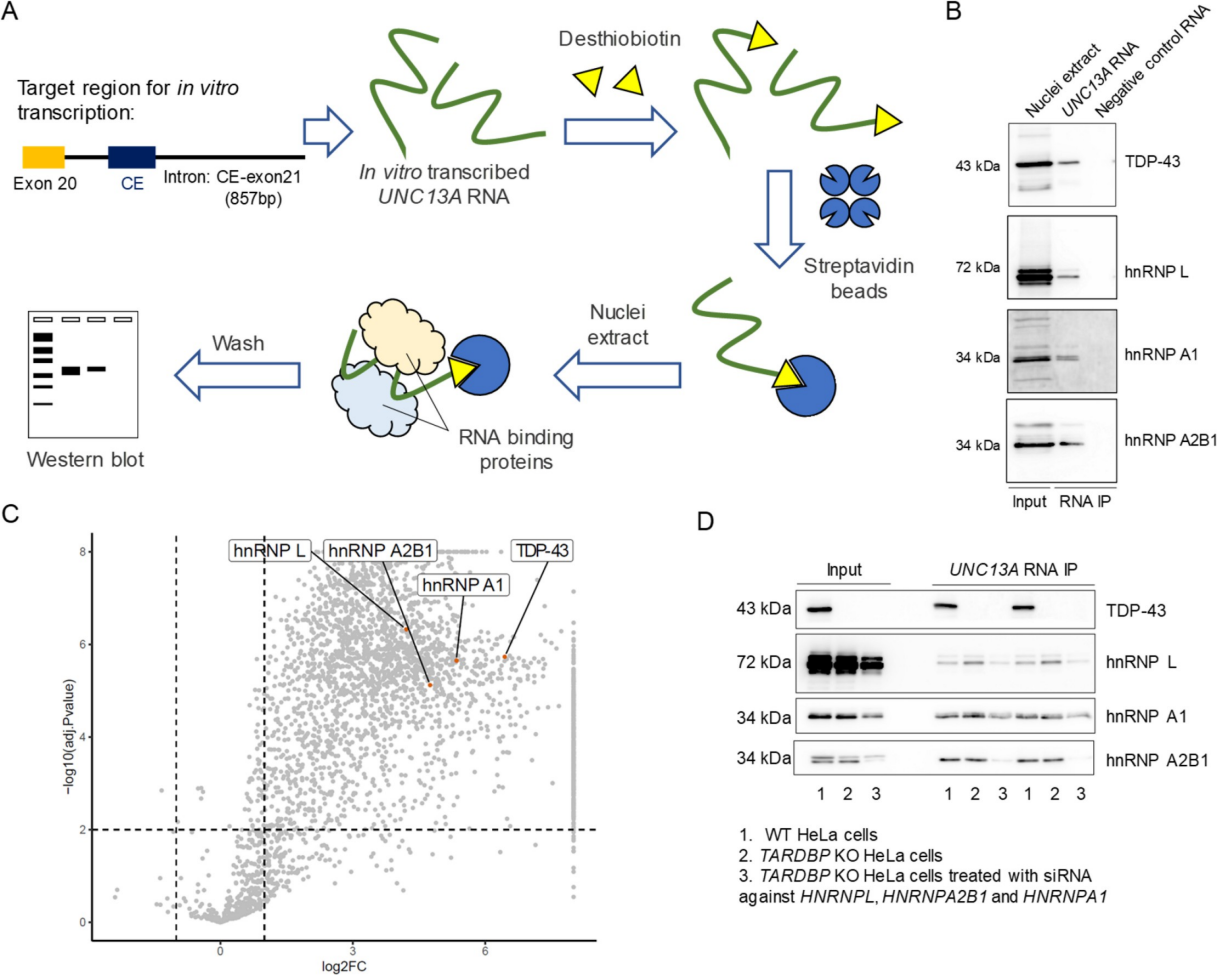
hnRNP L, hnRNP A1, and hnRNP A2B1 bind to *UNC13A* RNA independent of TDP-43. (**A**) Schematic representation of RNA pull-down system to identify proteins that bind *UNC13A* RNA. First, *UNC13A* RNA is in vitro transcribed from *UNC13A* minigene construct. Second, the RNA is labeled with a T4 RNA ligase, and the labeled *RNA* is then captured with streptavidin magnetic beads. The *UNC13A* RNA-streptavidin beads complex is mixed with either WT or *TARDBP* KO HeLa cell nuclei extract to elute the *UNC13A* RBPs, which are then assessed by western blot. (**B, C**) In vitro-transcribed RNA from WT *UNC13A* minigene (containing reference haplotype in *UNC13A*) showed binding to TDP-43, hnRNP L, hnRNP A1, and hnRNP A2B1 by western blot (**B**) and mass spectrometry (**C**). Blots in B provided in Supporting information (S1 Raw images). Data used to generate the volcano plot in C can be found in S1 Table. (**D**) In vitro-transcribed RNA from WT *UNC13A* minigene demonstrate binding of *UNC13A* cryptic exon to hnRNP L, hnRNP A1, and hnRNP A2B1 even in the absence of TDP-43 (*TARDBP* KO HeLa cells), as shown in western blot. Blots in D provided in Supporting information (S1 Raw images). *TARDBP* KO HeLa cells treated with siRNAs against *HNRNPL*, *HNRPNA1*, and *HNRNPA2B1* were used as an additional negative control in the assay. Representative images of at least 2 independent experiments are shown. RBP, RNA-binding protein; siRNA, small interfering RNA; TDP-43, TAR DNA-binding protein-43.

Given that other hnRNPs are also implicated in repression of cryptic exons [6,14–17], we investigated their ability to bind *UNC13A* RNA. In particular, we focused on hnRNPs involved in the regulation of *SORT1* cryptic splicing: hnRNP L, hnRNP A1, and hnRNP A2B1 [17]. To this end, we performed RNA pull-downs using in vitro transcribed RNA from the *UNC13A* WT minigene as bait and incubated with nuclear extracts (**Fig 3A**). In addition to TDP-43, hnRNP L, hnRNP A1, and hnRNP A2B1 were able to bind *UNC13A* RNA (**Fig 3B**). To confirm our findings and identify other hnRNPs involved in *UNC13A* splicing using an unbiased approach, we performed mass spectrometry following RNA pull-down. We identified several proteins, including hnRNP L, hnRNP A1, and hnRNP A2B1 that were significantly enriched in the presence of *UNC13A* RNA compared to a negative control RNA (**Fig 3C and S1 Table**). As anticipated, Gene Ontology (GO) enrichment analysis of *UNC13A* RNA-binding proteins (RBPs) revealed RNA metabolism, mRNA processing, and RNA splicing as the most significantly enriched biological processes (**Graph A in S2 Fig and S2 Table**). Significant enrichment for terms relating to RNA binding were found for molecular function analysis (**Graph B in S2 Fig and S2 Table**), and nuclear body and spliceosome were significantly enriched for cellular compartment analysis (**Graph C** in **S2 Fig and S2 Table**). Similarly, KEGG pathway analysis revealed significant enrichment of the spliceosome and mRNA surveillance pathways (**Graph D in S2 Fig and S2 Table**). Despite identifying many other proteins that bind *UNC13A* RNA, we focused our subsequent efforts on our initial 3 candidates, given their involvement in the regulation of cryptic splicing of TDP-43 targets [17]. TDP-43 is reported to form protein–protein interactions with both hnRNP A1 and hnRNP A2B1 [7,36]. Therefore, to evaluate whether the interaction of hnRNP L, hnRNP A1, and hnRNP A2B1 with *UNC13A* RNA is dependent or independent of TDP-43, we examined their binding ability in *TARDBP* KO cells. RNA pull-down assays revealed that hnRNP L, hnRNP A1, and hnRNP A2B1 bind similarly to *UNC13A* RNA in the presence or absence of TDP-43 (**Fig 3D**). Binding of hnRNP L, A1 and A2B1 were reduced in *TARDBP* KO HeLa cells when all 3 hnRNPs were down-regulated (**Fig 3D**). Of note, the protein levels of hnRNP L, hnRNP A1, and hnRNP A2B1 were similar between WT and *TARDBP* KO cells (**S3 Fig**). Overall, these data provide evidence that multiple hnRNPs can bind *UNC13A* RNA independently of TDP-43.

### The presence of the risk haplotype in *UNC13A* cryptic exon decreases the binding ability of hnRNP L, hnRNP A1, and hnRNP A2B1 to *UNC13A* RNA

**Fig 4:**
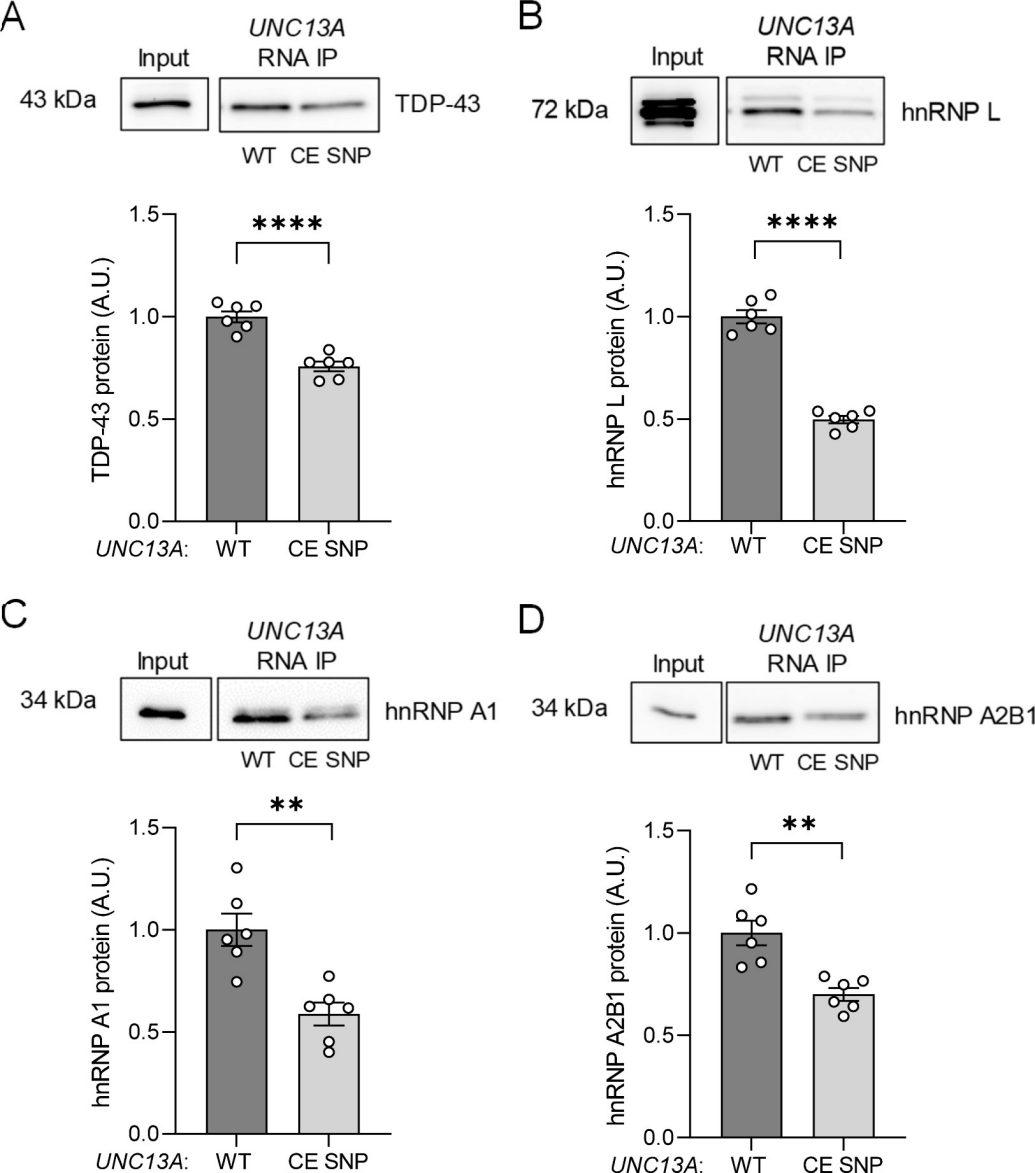
The presence of the risk haplotype in *UNC13A* cryptic exon affects its binding ability to hnRNP L, hnRNP A1, and hnRNP A2B1. In vitro-transcribed RNA from WT and CE SNP *UNC13A* minigenes were incubated with nuclear extracts from WT HeLa cells to assess their ability to bind the following proteins by western blot analyses after pull-down: TDP-43 (**A**), hnRNP L (**B**), hnRNP A1 (**C**), and hnRNP A2B1 (**D**). The graphs show reduced binding to CE SNP minigene by TDP-43 and other hnRNPs, as quantified by the signal intensity of the western blots using Image J. Graphs represent mean ± SEM of 6 independent assays. Statistical differences were assessed by Student’s *t* test, **P < 0.005, ****P < 0.0001. Blots provided in Supporting information (S1 Raw images). Data used to generate graphs can be found in S3 Table. CE, cryptic exon; SEM, standard error of mean; SNP, single-nucleotide polymorphism; TDP-43, TAR DNA-binding protein-43.

Previous studies revealed that TDP-43 has decreased binding affinity for RNA with the risk haplotype located in *UNC13A* cryptic exon [21]. To determine if the binding of other hnRNPs was perturbed as a function of the *UNC13A* CE SNP, we performed RNA pull-down assays using RNA from *UNC13A* minigenes harboring either the reference haplotype (WT) or the risk haplotype within the cryptic exon (CE SNP) as bait. As anticipated, western blotting following the pull-down, revealed significantly lower levels of TDP-43 bound to the *UNC13A* CE SNP compared to *UNC13A* WT (**Fig 4A**). Similarly, hnRNP L (**Fig 4B**), hnRNP A1 (**Fig 4C**), and hnRNP A2B1 (**Fig 4D**) all demonstrated significantly diminished ability to bind to the *UNC13A* CE SNP, with hnRNP L showing the largest reduction in binding. We also performed UV-crosslinking and RNA-hnRNP L immunoprecipitation (CLIP) in *TARDBP* KO HeLa cells expressing the *UNC13A* WT minigene, confirming the interaction of hnRNP L with *UNC13A* cryptic RNA (**S4 Fig**). To identify potential RNA-binding sites within the *UNC13A* cryptic exon and surrounding intronic sequences, we queried a database containing known RNA binding motifs [37]. Binding sites for hnRNP A2B1 were predicted within the *UNC13A* cryptic exon and within the intronic flanking regions for hnRNP A2B1, hnRNP A1, and hnRNP L (**S5 Fig**). Since hnRNP L binding was decreased in the presence of the SNP, we reasoned it likely binds at or near the cryptic exon. To test this, we generated an *UNC13A* minigene construct lacking the cryptic exon sequence (ΔCE, **Schematic A in S6 Fig**) and performed RNA pull-down assays. hnRNP L showed reduced binding activity to the *UNC13A* ΔCE, compared to the *UNC13A* WT construct (**Data B in S6 Fig**), supporting that hnRNP L likely interacts with *UNC13A* RNA near the cryptic exon. Together, these data suggest there is a global reduction in the ability of hnRNPs to bind *UNC13A* RNA with the risk haplotype located within the cryptic exon.

### In the absence of TDP-43, hnRNP L, hnRNP A1, and hnRNP A2B1 can repress *UNC13A* cryptic exon inclusion

**Fig 5:**
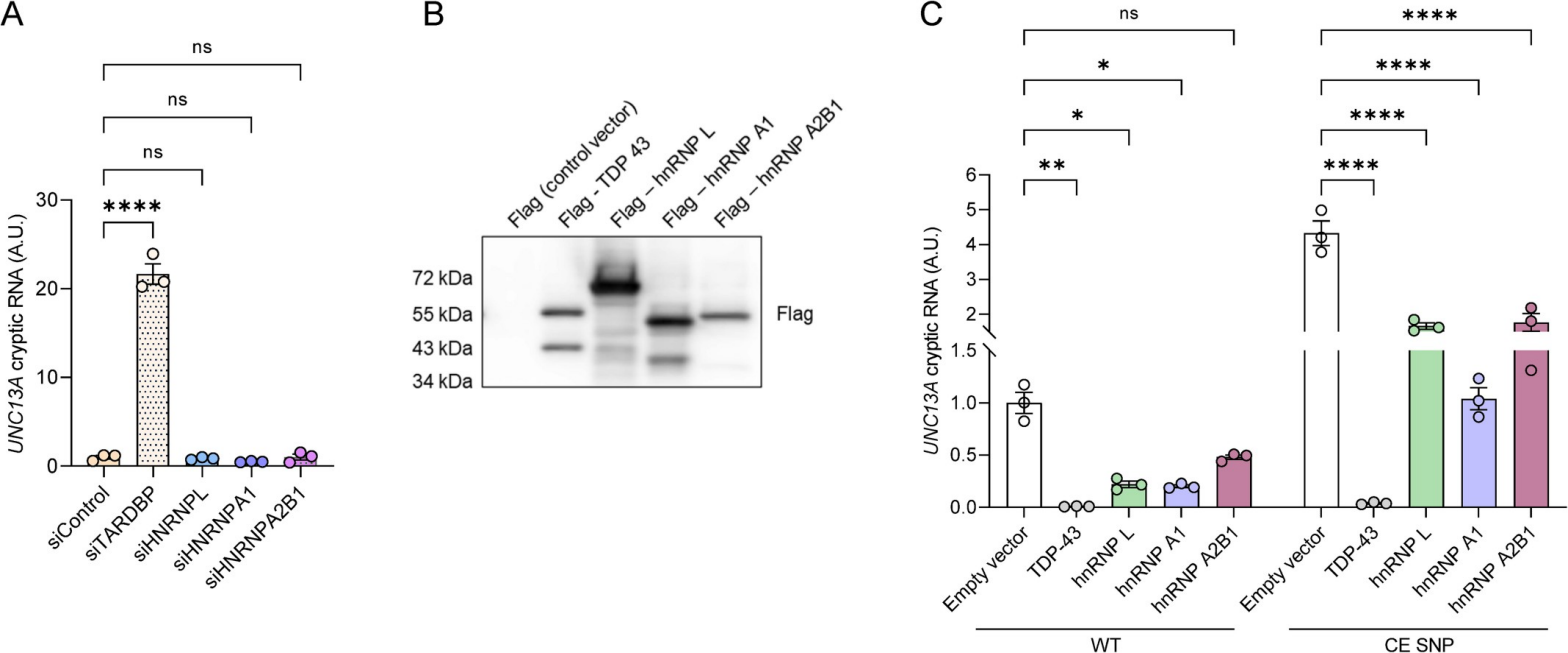
hnRNP L, hnRNP A1, and hnRNP A2B1 can repress *UNC13A* cryptic exon splicing but TDP-43 down-regulation is critical to observe *UNC13A* cryptic RNA accumulation. (**A**) WT *UNC13A* minigene was expressed in WT HeLa cells treated with either control (siControl) or siRNAs against *TARDBP* (siTARDBP), *HNRNPL* (siHNRPL), *HNRNPA1* (siHNRNPA1), or *HNRNPA2B1* (siHNRNPA2B1). RNA was extracted, and RT-qPCR was performed to assess the expression levels of *UNC13A* cryptic (A), *TARDBP* (S7A Fig), *HNRNPL* (S7B Fig), *HNRNPA1* (S7C Fig), or *HNRNPA2B1* (S7D Fig) RNA. (B, C) Flag-tagged TDP-43, hnRNP L, hnRNP A1, and hnRNP A2B1 were expressed in *TARDBP* KO HeLa cells transfected with *UNC13A* WT or CE SNP minigenes to evaluate the ability of other hnRNPs to repress *UNC13A* cryptic exon inclusion by RT-qPCR. A representative immunoblot confirming the expression of each Flag-tagged plasmid using a Flag antibody is shown in B. Blot provided in Supporting information (S1 Raw images). All graphs represent mean ± SEM of *UNC13A* cryptic RNA levels of 3 independent experiments. Statistical differences were assessed by one-way followed by Tukey’s multiple comparisons test (A) or two-way (C) ANOVA (ns: not significant, *P < 0.05, **P < 0.005, ****P < 0.0001). Data used to generate graphs can be found in S3 Table. CE, cryptic exon; hnRNP, heterogeneous nuclear ribonucleoprotein; SEM, standard error of mean; siRNA, small interfering RNA; SNP, single-nucleotide polymorphism;TDP-43, TAR DNA-binding protein-43.

Given that hnRNP L, hnRNP A1, and hnRNP A2B1 are able to bind *UNC13A* RNA, we wondered if they were also involved in regulating *UNC13A* splicing. Knockdown of TDP-43 (siTARDBP), but not of hnRNP L (siHNRNPL), hnRNP A1 (siHNRNPA1), or hnRNP A2B1 (siHNRNPA2B1), was sufficient for *UNC13A* cryptic RNA to accumulate in WT HeLa cells (**Figs 5A and S7**). Yet under conditions of TDP-43 depletion (*TARDBP* KO cells) knockdown of hnRNP L resulted in significantly elevated levels of *UNC13A* cryptic RNA harboring the reference haplotype (WT, **S8 Fig**). Overall levels of cryptic exon inclusion were elevated with expression of the *UNC13A* CE SNP minigene compared to WT minigene, but no differences were observed following knockdown of hnRNP L or hnRNP A2B1 (**S8 Fig**).

Next, we assessed the ability of TDP-43, hnRNP L, hnRNP A1, and hnRNP A2B1 to rescue *UNC13A* splicing by co-transfecting constructs expressing hnRNPs with either the *UNC13A* WT or CE SNP minigene in *TARDBP* KO cells (**Fig 5B and 5C**). All hnRNPs were well expressed in *TARDBP* KO cells (**Fig 5B**). As shown earlier, restoring TDP-43 protein expression rescued *UNC13A* splicing, even in the presence of the *UNC13A* risk haplotype (**Fig 5C**). More importantly, expression of hnRNP L, hnRNP A1, or hnRNP A2B1 was able to partially rescue *UNC13A* splicing (**Fig 5C**). Together, these results confirm TDP-43 is the primary repressor of the *UNC13A* cryptic exon. However, augmenting the levels of hnRNP L, hnRNP A1, and hnRNP A2B1 can partially rescue *UNC13A* splicing in the context of TDP-43 loss of function.

### Higher levels of hnRNP L protein associate with reduced *UNC13A* cryptic exon inclusion in FTLD-TDP

**Fig 6:**
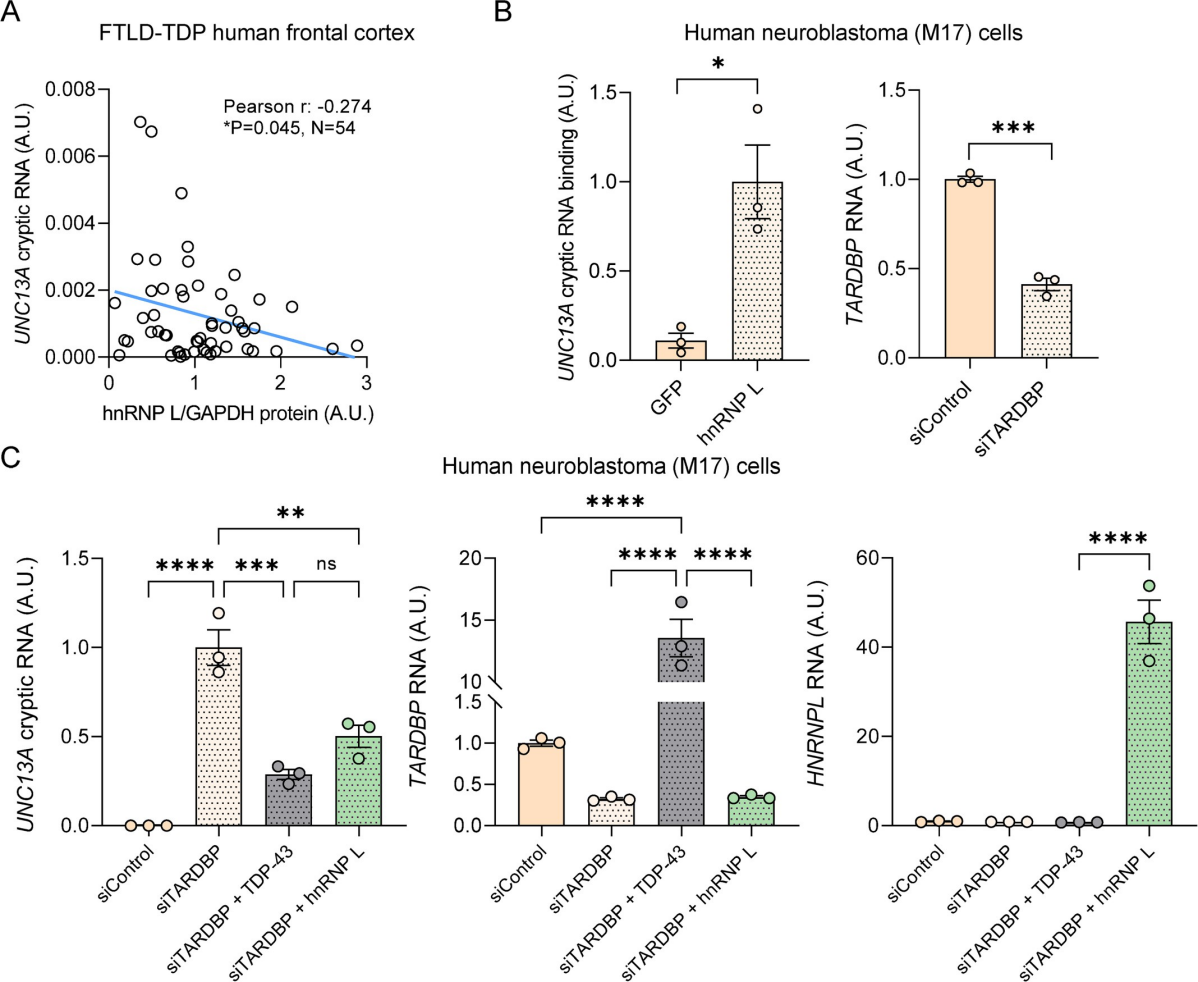
hnRNP L protein levels associate with *UNC13A* cryptic RNA accumulation in FTLD-TDP cases, and hnRNP L can bind and repress *UNC13A* cryptic exon splicing in human neuroblastoma (M17) cells upon TDP-43 down-regulation. (**A**) hnRNP L protein levels were measured in frontal cortex samples from 54 FTLD-TDP cases by western blot and quantified by Image J. The association of hnRNP L protein levels with *UNC13A cryptic* RNA using Pearson correlation test is shown. (**B**) Human M17 cells were transfected with siRNA targeting *TARDBP* 3′ UTR. Following transfection, cells were UV-irradiated, and hnRNP L-bound RNA was immunoprecipitated using a mouse monoclonal hnRNP L antibody [4D11] (ab6106, Abcam) as explained in Materials and methods. GFP immunoprecipitation served as negative control in the assay. qRT-PCR analysis demonstrates endogenous *UNC13A* RNA bound to hnRNP L but not GFP. (**C**) qRT-PCR of *UNC13A* cryptic RNA demonstrates the ability of Flag-tagged TDP-43 and hnRNP L to repress endogenous *UNC13A* mis-splicing. Levels of *TARDBP* and *HNRNPL* RNA were also evaluated to verify their expression. Graphs in **B** and **C** represent mean ± SEM of 3 independent replicates. Statistical differences were assessed by Student’s *t* test (**B**) or one-way ANOVA followed by Tukey’s multiple comparisons test (**C**) (ns: not significant, *P < 0.05, **P < 0.005, ***P < 0.0005, ****P < 0.0001). Data used to generate graphs can be found in S3 Table. hnRNP, heterogeneous nuclear ribonucleoprotein; qRT-PCR, quantitative reverse transcription polymerase chain reaction; SEM, standard error of mean; siRNA, small interfering RNA; TDP-43, TAR DNA-binding protein-43.

Given that our data suggested than hnRNP L, hnRNP A1, and hnRNP A2B1 repress *UNC13A* splicing in TDP-43 depleted cells, we wondered whether there was evidence of a relationship between these events in FTLD-TDP. To this end, we measured hnRNP L, hnRNP A1, and hnRNP A2B1 protein levels in the frontal cortex, a tissue with high burden of TDP-43 pathology and TDP-43 nuclear clearance, of 54 FTLD-TDP cases, and compared to the levels of *UNC13A* cryptic RNA in the same samples. No significant associations were found between the levels of hnRNP A1 or hnRNP A2B1 and *UNC13A* cryptic RNA (**S9 Fig**). Intriguingly, we found a significant correlation between higher hnRNP L protein levels and a lower burden of *UNC13A* cryptic exon inclusion (**Fig 6A**). To determine if hnRNP L can bind and regulate *UNC13A* cryptic RNA splicing in a physiological relevant cell type, we performed CLIP of hnRNP L-bound RNAs in human neuroblastoma (M17) cells in which TDP-43 has been down-regulated using siRNA targeting *TARDBP* (siTARDBP, **Fig 6B**). Analysis by qRT-PCR demonstrated that hnRNP L binds the endogenous *UNC13A* cryptic transcript (**Fig 6B**). Moreover, to determine whether hnRNP L can rescue *UNC13A* cryptic splicing in a neuronal-like cell, we also overexpressed hnRNP L and TDP-43 in M17 cells in which TDP-43 was knocked down using siRNA that targets the 3′ UTR and thus will not interfere with TDP-43 overexpression (**Fig 6C**). Like TDP-43, hnRNP L was able to repress the accumulation of endogenous *UNC13A* cryptic RNA (**Fig 6C**). These results suggest that hnRNP L may be able compensate for TDP-43 loss of function by regulating the splicing *UNC13A*.

## Discussion

UNC13A is emerging as a key player in ALS/FTD pathogenesis. Loss of the mouse UNC13A homologue, Munc13-1, leads to perinatal lethality, likely due to its essential role in synaptic vesicle maturation and neurotransmitter release from glutamatergic neurons [22–25,38]. Recent studies have demonstrated that TDP-43 is critical for *UNC13A* splicing, and its depletion results in a reduction of UNC13A protein [20,21]. Thus, insight into the regulation of *UNC13A* splicing is crucial to identify targets for therapeutic intervention. Here, we explore the contribution of hnRNPs to *UNC13A* splicing.

We first examined whether dimerization of TDP-43 is critical for binding of *UNC13A* RNA and cryptic exon repression. We found that TDP-43 mutations in the extreme N-terminus (TDP-43_N-term del/mut_), which not only disrupt TDP-43 dimer formation but also reduce TDP-43 stability [34], are unable to fully rescue *UNC13A* cryptic exon inclusion compared TDP-43_WT_. This is likely a result from reduced binding activity of these mutants. Interestingly, more subtle disruption of TDP-43’s N-terminal domain polymeric interactions (TDP-43_E17R_, [35]) did not affect its ability to bind *UNC13A* RNA, and TDP-43_E17R_ was able to repress inclusion of the *UNC13A* cryptic exon similarly to TDP-43_WT_. This was somewhat unexpected given that previous studies reported TDP-43_E17R_ mutant to have reduced splicing activity for another target, the *CFTR* gene [35]. These differences may be due to different experimental systems or targets. Nonetheless, our findings that deletion or mutation of multiple residues of TDP-43’s N-terminal domain affect spicing activity are consistent with reports for other TDP-43 targets [34,38]. Taken together, these data indicate that TDP-43 binding to *UNC13A* is required for maximal splicing activity, and certain perturbations to TDP-43 N-terminal domain may also impact splicing activity.

We find, both with and without TDP-43 present, hnRNP L, hnRNP A1, and hnRNP A2B1 bind *UNC13A* RNA, suggesting that a network of hnRNPs is involved in binding *UNC13A* RNA, reminiscent of our findings that multiple hnRNPs bind *SORT1* RNA [17]. hnRNP A1 and hnRNP A1B2 have previously been shown to bind to the C-terminal domain of TDP-43 [36,39]. Here, we find these proteins can bind to *UNC13A* RNA independently of TDP-43. Further studies are needed to clarify if these interactions are indirect, a result of other protein–protein interactions, or if hnRNPs directly bind to *UNC13A* RNA, and to which RNA motifs they bind. Our unbiased analysis by mass spectrometry revealed several proteins can bind *UNC13A* RNA in addition to our 3 candidates. Other hnRNPs emerging from our studies could potentially regulate *UNC13A* splicing; therefore, more exhaustive studies are needed to fully define and validate the repertoire of hnRNPs involved in *UNC13A* splicing and cryptic exon repression. Additionally, we find the presence of the risk haplotype within the *UNC13A* cryptic exon decreases the binding affinity of hnRNP L, hnRNP A1, and hnRNP A1B2, suggesting that the risk SNP alters the entire network of hnRNPs involved in splicing regulation.

Loss of TDP-43 alone is sufficient to induce accumulation of the *UNC13A* cryptic RNA, confirming that it is the primary regulator of *UNC13A* cryptic exon repression. However, in the absence of TDP-43, down-regulation of hnRNPs L further enhanced *UNC13A* cryptic RNA accumulation in the context of TDP-43 loss of function, and increasing the levels of hnRNP L, hnRNP A1, and hnRNP A2B1 reduced the accumulation of *UNC13A* cryptic RNA. Most importantly, we find evidence, in human disease, that higher protein levels of hnRNP L, but not hnRNP A1 or hnRNP A2B1, correlate with lower levels of *UNC13A* cryptic RNA accumulation in the frontal cortex of FTLD-TDP cases. Further, we demonstrated an endogenous interaction of hnRNP L binding to and repressing cryptic exon inclusion in *UNC13A* RNA in human neuronal cells. Together, our findings suggest that hnRNP L represses *UNC13A* cryptic exon inclusion and compensates for TDP-43 loss of function both in cells and in human disease.

We have previously found that hnRNP L can regulate the splicing of the TDP-43 target, *SORT1* [17], silencing of hnRNP L in *Drosophila* neurons, alone or in combination with TDP-43 fly ortholog, led to severe locomotor defects, signaling a genetic interaction of these 2 proteins that illicit ALS linked phenotypes [40]. hnRNP L has been reported to repress cryptic exons in various target genes by binding to CA-rich repeats or clusters [14,41,42], and indeed our RNA binding motif analyses identified hnRNP L binding motifs within the *UNC13A* intron. However, we found that hnRNP L binding is decreased in constructs with the cryptic exon deleted, suggesting that hnRNP L likely binds within the cryptic exon itself. Future studies should evaluate whether hnRNP L may bind to specific motifs within or surrounding the *UNC13A* cryptic exon or alternatively, exist in a complex with other hnRNPs. Moreover, identification of which domains within hnRNP L are required to *UNC13A* cryptic RNA binding should also be evaluated. Thus, given our findings that hnRNP L levels correlate with *UNC13A* cryptic exon repression, further study of the involvement of hnRNP L in regulating *UNC13A* and other TDP-43 targets are warranted.

Beyond hnRNP L, multiple hnRNPs and their role in splicing have already been implicated in ALS/FTD. Recently, Bampton and colleagues showed that hnRNP K mis-localizes in FTLD-TDP brains with increased transcripts with cryptic exons [16]. PTBP1 splicing activity is dysregulated in FTLD-TDP brains [43]. Highlighting, the importance of hnRNPs and splicing activity, mutations in the low complexity domains of hnRNP A2B1 and hnRNP A1 have been found casual for ALS [44], with mutations in hnRNP A2B1 producing widespread splicing changes in fibroblasts and motor neurons [45]. We now implicate other hnRNPs in the splicing of *UNC13A* RNA, compensating for TDP-43 in its absence.

The complex mechanisms of how TDP-43 and other hnRNPs co-regulate targets are just emerging. Indeed, several hnRNPs can bind the RNA targets of TDP-43 and regulate their splicing [17,40,46], and RNA levels of certain hnRNPs are elevated in FTLD-TDP [17]. TDP-43’s role in the repression of cryptic exons had been shown to be cell-type specific, with unique targets identified in neurons, stems cells, muscle cells, Schwann cells, and oligodendrocytes [8,12,47–49]. Susnjar and colleagues have proposed differential expression of RBPs in cells and tissue may mediate the variability of TDP-43 targets [50]. They demonstrate that knocking down RBPs characteristic to a particular tissue could affect TDP-43-regulated splicing, suggesting that co-regulation between TDP-43 and other RBPs is required for target specificity [50]. Multiple hnRNPs directly play a role in cryptic exon suppression, understanding their cell-type specific effects and the overlap with TDP-43 will provide increased understanding to their role in ALS/FTD pathogenesis. Further exploration into whether and to what extent hnRNPs are acting independently or co-operatively will also be of great importance. These studies will obviously be challenging as TDP-43 binds to and regulates the splicing of other hnRNPs, like hnRNP A1. In the absence of TDP-43, an hnRNP A1 variant with increased aggregation, and toxicity is generated, and this variant is mis-localized to the cytoplasm within inclusions in ALS cases [51].

Our study significantly expands our knowledge of the factors that regulate cryptic exon inclusion in *UNC13A*, an important TDP-43 target gene. Our data suggests the landscape of hnRNPs, and specifically hnRNP L, are ALS/FTD disease modifiers, acting to limit aberrant splicing events by compensating for TDP-43 when it is depleted.

## Methods

### Human subject characteristics

Postmortem frontal cortex samples from patients with neuropathologically confirmed FTLD-TDP were obtained from the Brain Bank for Neurodegenerative Disorders at Mayo Clinic Florida. Autopsies were performed after consent by the next-of-kin or someone with legal authority to grant permission. The Brain Bank operates under protocols approved by the Mayo Clinic Institutional Review Board (IRB). A total of 54 FTLD-TDP cases were included in this study. The median age at death was 68 years (range: 51 to 90 years), and median age of onset was 63 years (range: 44 to 78 years) with a median disease duration of 6 years (range: 1 to 16 years). Note age at onset and survival information was not available for 3 cases. The cohort included both males (*N* = 31, 57.4%) and females (*N* = 23, 42.6%), and all cases were carriers of either *C9orf72* (*N* = 46) or *GRN* (*N* = 8) mutations.

### Cell culture

Parental (wild-type, WT) HeLa cell line (human cervix carcinoma, female, from ATCC) and a monoclonal *TARDBP* CRISPR-depleted HeLa cell line (*TARDBP* HeLa KO cells), a generous gift from Dr. Shawn Ferguson [32], were grown in DMEM medium (Gibco) plus 10% fetal bovine serum (Sigma) and 1% penicillin/streptomycin (Gibco). M17 cell line (human neuroblastoma, from ATCC) was grown in Opti-MEM I + GlutaMax I medium (Gibco) plus 10% fetal bovine serum (Sigma) and 1% penicillin/streptomycin (Gibco).

### Generation of *UNC13A* minigene constructs

The *UNC13A* minigene construct containing the human *UNC13A* cryptic exon sequence and the nucleotide flanking sequences upstream (50 bp at the of end of intron 19, the entire exon 20, the entire intron 20 sequence upstream of the cryptic exon) and downstream (remaining 857 bp downstream sequence of intron 20) of the cryptic exon were amplified from human genomic DNA using the following primers: 5′AGGTCATATGCACTGCTATAGTGGGAAGTTC and 5′-CTTACATATGGCCACCATGGGAGAGAAAG, and subcloned into the *NdeI* site of the pTB vector, which was kindly provided by Dr. Emanuele Buratti. Minigenes containing the risk haplotypes were made using the WT (reference haplotype) minigene as a template for site-directed mutagenesis using the QuikChange II XL Site-Directed Mutagenesis Kit (Agilent), according to the manufacturer’s directions and the following primers: 5′-CCCATCTCTCCATCCATGCTTTTATCTACTCATCACT and 5′-AGTGATGAGTAGATAAAAGCATGGATGGAGAGATGGG for rs12973192; 5′-ACAGACGAAAAATGGATGGGTGGATAAATTGATGGGTGG and 5′-CCACCCATCAATTTATCCACCCATCCATTTTTCGTCTGT for rs12608932.

To generate constructs for RNA pull-down experiments, the following primers were used and cloned into pcDNA6 V5 His A vector (Invitrogen): 5′-AGCCAAGCTTACAAGCGAACTGACAAATCTG and 5′-ACCTCTCGAGGCCACCATGGGAGAGAAAG. The *UNC13A* gene fragment construct lacking the cryptic exon were amplified from the *UNC13A* minigene constructs using the primers listed above, as well as the following primers 5′-CATTGGTCTCCCTGGAAGAGACATACCC and 5′-AATGGGTCTCACCAGGTGAGTACATGGATG to clone it into the pcDNA6 V5 His A vector (Invitrogen) and using a Type IIS restriction enzyme.

### TDP-43 and other hnRNP overexpression constructs

Constructs to express GFP-tagged or Flag-tagged TDP-43 proteins (GFP-TDP-43_WT_ or Flag-TDP-43 _WT_) have been previously described [52,53]. Constructs to express GFP-tagged TDP-43 with RNA-binding mutations (GFP-TDP-43_5FL_), TDP-43 lacking the first 2–9 N-terminal residues (GFP-TDP-43_N-term del_) and TDP-43 bearing mutations to key N-terminal residues (R6G, V7G, T8G, E9G; GFP-TDP-43_N-term mut_) have been previously described [34]. GFP-TDP43_E17R_ mutant was generated using WT GFP-TDP-43 as a template and the QuikChange II XL Site-Directed Mutagenesis Kit (Agilent), according to the manufacturer’s directions and the following primers: 5′-CATCGTCTTCCGATGGTATTCTAATGGGCTCATCGTTCTCAT and 5′-ATGAGAACGATGAGCCCATTAGAATACCATCGGAAGACGATG. To generate Flag-tagged hnRNP A1 and hnRNP A2B1 overexpression constructs, an hnRNP A2B1 protein vector (pPM-N-D-C-HA) (Applied Biological Materials, Accession Number BC000506) and an hnRNP A1 protein vector (pPM-N-D-C-HA) (Applied Biological Materials, Accession Number BC002355) were used. The Flag-tagged hnRNP L overexpression construct (pPM-N-D-C-HA HNRNP L) was generated using pPM-N-D-C-HA HNRNPA2B1 protein vector after excising the hnRNP A2B1 coding sequence. The coding sequence of hnRNP L was amplified from a plasmid (Sino Biological, HG18369-U) using the Kapa Hi Fi PCR Kit (Roche) and the following primers: 5′-ATTCGTTTAAACTTATGCCTAAAAAGAGACAAGCAC and 5′GTCATCTAGAGGAGGCGTGCTGAGCAG, then cloned into the above vector backbone.

### Overexpression of TDP-43 or other hnRNPs to assess *UNC13A* splicing repression ability

To assess the ability of TDP-43 variants or other hnRNPs on regulating *UNC13A* splicing, *TARDBP* CRISPR-depleted (*TARDBP* KO) HeLa cells were co-transfected with 1.0 μg of the indicated *UNC13A* minigene constructs (WT: reference haplotype, CE SNP: risk haplotype in CE, intron SNP: risk haplotype in intron, or CE + intron SNP: risk haplotype in CE + intron) and 1.0 μg of one of the following plasmids: GFP, GFP-TDP-43_WT_, GFP-TDP-43_5FL_, GFP-TDP-43_N-term del_, GFP-TDP-43_N-term mut_, GFP-TDP-43_E17R_, Flag-empty vector, Flag-TDP-43_WT_, Flag-hnRNP L, Flag-hnRNP A1, Flag-hnRNP A2B1 constructs using Lipofectamine 2000 following manufacturer’s instructions (Invitrogen), for 48 h. To evaluate the ability of TDP-43 or hnRNPs on repressing splicing of endogenous *UNC13A* cryptic RNA, M17 cells were transfected with 1.0 μg of one of the following plasmids: Flag-empty vector, Flag-TDP-43_WT_ or Flag-hnRNP L constructs using Lipofectamine 2000 following manufacturer’s instructions (Invitrogen). Four hours following transfection, cells were treated with siLentfect (Bio-Rad) and siRNA complexes: AllStars Neg. Control siRNA (Cat#1027281, QIAGEN) or siRNA against *TARDBP* 3′ UTR, a region not included in the TDP-43 overexpression constructs (DNA target sequence: 5′-AAGAGTTGTCATTGTTGGAAA, QIAGEN) following manufacturer’s instructions, and incubated for 48 h. Cycloheximide (Sigma) was added to HeLa and M17 cells at a final concentration of 100 μg/ml at 6 h prior harvesting the cells. All experiments were done in triplicate.

### Knockdown of *TARDBP* and other *HNRNPs*

In knockdown experiments using WT or *TARDBP* KO HeLa cells, cells were incubated with siLentfect (Bio-Rad) and siRNA complexes: AllStars Neg. Control siRNA (QIAGEN, control for TDP-43 knockdown assay, Cat# 1027281), siGENOME Control Pool Non-Targeting (Dharmacon, control for other hnRNPs knockdown assay, Cat# D-001206-13-20), siRNA against *TARDBP* 3′ UTR (DNA target sequence: 5′-AAGAGTTGTCATTGTTGGAAA, QIAGEN), or siRNA against *HNRNPL* (L-011293-01-0005, Dharmacon), *HNRNPA1* (L-008221-00-0005, Dharmacon) or *HNRNPA2B1* (L-011690-01-0005, Dharmacon) following manufacturer’s instructions for 48 h. All experiments were replicated 3 or 4 times.

### RNA extraction, cDNA synthesis, and qPCR

Cultured cells were harvested and RNA extracted using TRIzol Reagent (Zymo Research), following manufacturer’s instructions. A total of 2.0 μg of RNA was converted into cDNA using the High Capacity cDNA Reverse Transcription Kit with RNA inhibitor (Applied Biosystems). The qRT-PCR assay was performed on cDNA (diluted 1:40) with SYBR GreenER qPCR SuperMix (Invitrogen) using QuantStudio7 Flex Real-Time PCR System (Applied Biosystems). All samples were analyzed in triplicates. The qRT-PCR program was as follows: 50°C for 2 min, 95°C for 10 min, and 40 cycles of 95°C for 15 s and 60°C for 1 min. Relative quantification was determined using the ΔΔCt method and normalized to the endogenous controls *RPLP0* and *GAPDH*. The following primer pairs were used: 5′-GATTGAACAGATGAATGAGTGATGA and 5′TGTCTGGACCAATGTTGGTG for evaluation of *UNC13A* cryptic RNA in HeLa cells overexpressing *UNC13A* minigene constructs; 5′-TGGATGGAGAGATGGAACCT and 5′-GGGCTGTCTCATCGTAGTAAAC for evaluation of endogenous *UNC13A* cryptic RNA in M17 cells; 5′-GTTCGACAGTCAGCCGCATC and 5′-GGAATTTGCCATGGGTGGA for *GAPDH*; 5′TCTACAACCCTGAAGTGCTTGAT and 5′-CAATCTGCAGACAGACACTGG for *RPLP0*; 5′-TGGACGATGGTGTGACTGCAA and 5′- AGAGAAGAACTCCCGCAGCTCA for *TARDBP*, 5′-TGTAATCCTTGTGGCCCTGT and 5′-ATCAGCCCCATTGAGAGAGG for *HNRNPL*, 5′-CCTGAGGAGCCATTTTGAGC and 5′-ATAGCTGCATCCACCTCCTC for *HNRNPA1*; 5′-TTTGGGGATGGCTATAATGG and 5′-CCATAACCGGGGCTACCT for *HNRNPA2B1*.

### Immunoprecipitation of *UNC13A* RNA bound to GFP-tagged TDP-43

*TARDBP* KO HeLa cells were transfected with 5.0 μg of *UNC13A* WT minigene construct and 5.0 μg of one of the following plasmids: GFP-TDP-43_WT_, GFP-TDP-43_5FL_, GFP-TDP-43_N-term del_, GFP-TDP-43_N-term mut_, or GFP-TDP-43_E17R_ using Lipofectamine 2000 (Invitrogen). Forty-eight hours later, cells were UV-irradiated on ice at 300 mJ/cm2 and harvested. Cells were lysed by 10 min incubation in hypotonic lysis buffer [10 mM Tris-HCl (pH 7.5), 10 mM NaCl, 2 mM EDTA, 0.5% Nonidet-P40] supplemented with SUPERase-In RNase Inhibitor (5 μL/mL; Thermo Fisher) and protease inhibitor mixture (1:100; Millipore). Then, lysates were supplemented with NaCl to 150 mM, incubated 5 min on ice, and spun at 2,300 × g for 5 min. Cell debris was discarded and supernatants were used as protein lysates in the following assay. Bicinchoninic acid assays (Pierce) were performed to measure total protein concentration, and 300 μg of protein lysates were used for immunoprecipitation with Protein G Dynabeads (Invitrogen). Rabbit polyclonal anti-GFP antibody (ab290, Abcam) diluted in NT2 wash buffer [50 mM Tris (pH 7.4), 150 mM NaCl, 0.05% Nonidet P-40] (1:1,000) was added to the Protein G Dynabeads and incubated with rotation for 15 min at room temperature. Then, Protein G Dynabeads-GFP antibody complexes were incubated with precleared protein lysates (30 min at 4°C) overnight at 4°C. Following overnight incubation, beads were washed 6 times by NT2 wash buffer and resuspended in 200 μL of NT2 wash buffer supplemented with SDS to 2.5% and incubated with 30 U of Proteinase K (Invitrogen) for 30 min at 55°C to eliminate protein. Immunoprecipitated RNA was extracted using TRIzol Reagent (Zymo Research), following manufacturer’s instructions. All obtained RNA was converted into cDNA using the High-Capacity cDNA Reverse Transcription Kit with RNA inhibitor (Applied Biosystems). The qRT-PCR assay was performed as described in “RNA extraction, cDNA synthesis, and qPCR for *UNC13A* cryptic splicing” section. The following primer pair was used to detect *UNC13A* cryptic RNA: 5′-CAGCCCTAACCACTCAGGATT and 5′-TCATCACTCATTCATCTGTTCAATC.

### Immunoprecipitation of *UNC13A* RNA bound to endogenous hnRNP L

*TARDBP* KO HeLa cells were transfected with 5.0 μg of *UNC13A* WT minigene construct using Lipofectamine 2000 (Invitrogen). M17 cells were incubated with siLentfect (Bio-Rad) and siRNA (siRNA against *TARDBP* 3′ UTR, DNA target sequence: 5′**-**AAGAGTTGTCATTGTTGGAAA) complexes. Forty-eight hours later, cells were UV-irradiated on ice at 300 mJ/cm2 and harvested. Cells were lysed by 10 min incubation in hypotonic lysis buffer [10 mM Tris-HCl (pH 7.5), 10 mM NaCl, 2 mM EDTA, 0.5% Nonidet-P40] supplemented with SUPERase-In RNase Inhibitor (5 μL/mL; Thermo Fisher) and protease inhibitor mixture (1:100; Millipore). Then, lysates were supplemented with NaCl to 150 mM, incubated 5 min on ice, and spun at 2,300 × g for 5 min. Cell debris was discarded and supernatants were used as protein lysates in the following assay. Bicinchoninic acid assays (Pierce) were performed to measure total protein concentration, and 300 μg (*TARDBP* KO HeLa cells) or 750 μg (M17 cells) of protein lysates were used for immunoprecipitation with Protein G Dynabeads (Invitrogen). Mouse monoclonal anti-hnRNP L antibody [4D11] (ab6106, Abcam) and mouse monoclonal anti-GFP antibody [C163] (33–2600, Invitrogen) were added to precleared protein lysates (1 μg of antibodies to 300 μg of protein lysate) and incubated overnight at 4°C. Then, protein lysate-antibody complexes were incubated with Protein G Dynabeads 4 h at 4°C. Following incubation, beads were washed 6 times by NT2 wash buffer [50 mM Tris (pH 7.4), 150 mM NaCl, 0.05% Nonidet P-40] and resuspended in 200 μL of NT2 wash buffer supplemented with SDS to 2.5% and incubated with 30 U of Proteinase K (Invitrogen) for 30 min at 55°C to eliminate protein. Immunoprecipitated RNA was extracted using TRIzol Reagent (Zymo Research), following manufacturer’s instructions. All obtained RNA was converted into cDNA using the High-Capacity cDNA Reverse Transcription Kit with RNA inhibitor (Applied Biosystems). The qRT-PCR assay was performed as described in “RNA extraction, cDNA synthesis, and qPCR for *UNC13A* cryptic splicing” section. The following primer pair was used to detect *UNC13A* cryptic RNA: 5′-CAGCCCTAACCACTCAGGATT and 5′-TCATCACTCATTCATCTGTTCAATC.

### *In vitro* transcription of *UNC13A* RNA and pull-down of *UNC13A* RNA-bound proteins

RNA was transcribed from PCR templates amplified from *UNC13A* minigene constructs (WT and CE SNP). A T7 promoter sequence (TAATACGACTCACTATAGGG) was added towards the 5′ end of the using primer that carried a T7 sequence. To linearize the plasmid, a restriction enzyme site for *XhoI* used. For transcription, 5 μg of DNA template was used for each sample with mMESSAGE mMACHINE T7 transcription kit following manufacturer’s instructions (Thermo Fisher). The synthesized RNA was purified using MEGAclear Transcription Clean-Up Kit (Thermo Fisher). A total of 10 μg of transcribed RNA was used for pull-down with Pierce Magnetic RNA-Protein Pull-Down Kit according to manufacturer’s instructions (Thermo Fisher). Nuclear extract used for the assay were prepared from WT or *TARDBP* KO HeLa cells (both with and without additional knockdown of *HNRNPL*, *HNRNPA1*, and *HNRNPA2B1*) using a nuclear extraction kit (ab113474, Abcam) according to manufacturer’s protocol. Bicinchoninic acid assays (Pierce) were performed to measure total protein concentration, and 5 μg of protein was used for positive control for western blot analysis (input). Then, 2× SDS-loading buffer was applied, the kit-associated beads were gently sedimented, and the supernatant was removed for western blot analysis.

### Processing of cultured cells and human postmortem tissues for western blot analysis

Cultured cells were lysed by sonicating twice in lysis buffer [50 mM Tris-HCl (pH 7.4), 5 mM EDTA, 300 mM NaCl, 1% Triton X-100, protein inhibitor cocktail (Millipore) 1:100, PMSF (Sigma) 1:100, Phosphatase inhibitor cocktail A (bimake.com) 1:100, Phosphatase inhibitor cocktail B (bimake.com) 1:100]. Bicinchoninic acid assays (Pierce) were performed to measure protein concentration, and 10 μg of total protein was analyzed by immunoblotting for the proteins described below.

Approximately 50 mg postmortem tissue from the frontal cortex of FTLD-TDP cases were homogenized in cold RIPA buffer [25 mM Tris-HCl (pH 7.6), 150 mM NaCl, 1% sodium deoxycholate, 1% Nonidet P-40, 0.1% sodium dodecyl sulfate, and protease and phosphatase inhibitors]. Homogenates were centrifuged at 100,000 × *g* for 30 min at 4°C, and the supernatant was collected. Bicinchoninic acid assays (Pierce) were performed to measure protein concentration, and 20 μg of total protein was analyzed by immunoblotting for hnRNP protein levels as described below.

### Western blot analysis

Protein lysates were loaded into 4% to 20% Tris–glycine gels (Novex) with 125 V for 2 h and transferred to 0.45 μm nitrocellulose blotting membrane (Amersham) with 300 mA for 2 h. After transfer, blots were blocked with 5% nonfat dry milk in Tris-buffered saline −0.1% Triton X-100 (TBST) for 1 h, then incubated with mouse monoclonal GFP antibody (1:1,000, [C163], 33–2600, Invitrogen), rabbit polyclonal TDP-43 C-terminal antibody (1:1,000, 12892-1-AP, Proteintech), mouse monoclonal hnRNP L (1:200 in RNA pull-down assay, 1:1,000 in all other assays, [4D11] ab6106, Abcam), mouse monoclonal hnRNP-A1 antibody (1:500 in RNA pull-down, 1:1,000 in all other assays, sc-32301, Santa Cruz Biotechnology), mouse monoclonal hnRNP A2B1 antibody (1:200 in RNA pull-down assay, 1:1,000 in all other assays, [B-7] sc-374053, Santa Cruz Biotechnology), mouse monoclonal Flag antibody (1:1,000, clone M2, F3165, Sigma), or mouse monoclonal GAPDH antibody (1:5,000, H86504M, meridian bioscience) overnight at 4°C. Membranes were washed in 1× TBST, then incubated with donkey anti-rabbit or anti-mouse IgG conjugated to horseradish peroxidase (1:5,000; Jackson ImmunoResearch) for 1 h, then washed again. The bands were detected using Western Lightning Plus-ECL, Chemiluminescent Substrate (Perkin Elmer) and visualized using Amersham ImageQuant 800 (GE Healthcare). In RNA pull-down assays, to enhance the signals, SuperSignal West Pico Chemiluminescent Substrate (Thermo Fisher Scientific) was added to Western Lightning Plus-ECL, Chemiluminescent Substrate to equal 10% of the total volume. Bands were quantified using ImageJ by analyzing pixel density, and protein levels were normalized to GAPDH as the protein loading control. Uncropped blots are provided in Supporting information (S1 Raw images). Data used to generate graphs can be found in **S3 Table**.

### hnRNP L, hnRNP A1, and hnRNP A2B1 motif analyses in *UNC13A* cryptic RNA

*UNC13A* cryptic exon (chr19:17,753,223–17,753,350, hg19) and cryptic exon with flanking intronic (chr19:17,752,366–17,753,653, hg19) sequences were queried in a database containing known RNA-binding motifs (http://rbpmap.technion.ac.il/) [37] to identify sequences within *UNC13A* where hnRNP L, hnRNP A1, and hnRNP A2B1 may bind. High stringency level settings were applied in which 2 thresholds are established: *p* value < 0.005 (significant hits) and *p* value < 0.01 (suboptimal).

### Sample preparation for mass spectrometry-based proteomics

RNA pull-down assay was performed as described in “In vitro transcription of *UNC13A* RNA and pull-down of *UNC13A* RNA-bound proteins” section. In brief, nuclear extract used for the assay were prepared from WT HeLa cells using a nuclear extraction kit (ab113474, Abcam) according to manufacturer’s protocol. A total of 10 μg of in vitro transcribed WT *UNC13A* and control (from the RNA pull-down kit) RNAs were used for pull-down using Pierce Magnetic RNA-Protein Pull-Down Kit, according to manufacturer’s instructions (Thermo Fisher). After the RNA pull-down step, protein-bound beads were washed 3 times with ice cold PBS and 1 time with 50 mM ammonium bicarbonate (pH: 8.5, Sigma). Then, captured proteins were directly digested on-beads using 100 μl of 2% Trypsin/Lys-C (MS grade) (Promega) on a thermomixer at 1,200 rpm and 37°C for 16 h. After incubation, 10 μl of 5% Pierce trifluoroacetic acid (sequencing grade) were added to the beads (Thermo Fisher Scientific). Samples were then frozen and shipped for downstream mass spectrometry analyses. The resulting peptides were completely dried on a speed vacuum device for 2 h. The dry peptides were reconstituted in 2% acetonitrile with 0.5% trifluoroacetic acid and normalized to a final concentration of 0.2 μg/μl using peptide measurement on a Nanodrop. A total of 5 μl of each of the 6 replicates per condition were subjected to mass spectrometry analysis.

### Mass spectrometry-based proteomics

Data independent acquisition (DIA) was used for liquid chromatography and tandem mass spectrometry (LC-MS/MS)-based proteomics. The peptides were separated on 50 cm nanoLC column (75 μm I.D., 2 μm C18 particle) using a 90 min effective gradient 2% to 35% liquid phase B (i.e., 5% DMSO in 0.1% formic acid in acetonitrile). The peptides were then injected to an ultra-high resolution Orbitrap Eclipse mass spectrometer. The full scan was included as a part of DirectDIA workflow as we previously described [54]. The DIA-isolation window was set to 8 m/z with 1 m/z overlapped in a range of 400 to 1,000 m/z, resulting in 75 windows per DIA scan, and the loop control was set to 3 s duration. The fragmentation was conducted by 30% collision energy in HCD, and data was acquired by Orbitrap with a 30k resolution.

### Proteomics database search and statistical analyses

Spectronaut software (v16.2) was used to assign the peaks to correct peptides by the DirectDIA workflow. The workflow allows a search of the DIA data against a FASTA reference file containing 20,401 protein entries mapped to the human reference genome obtained via The UniProt Consortium (UP000005640). The trypsin and/or lysC enzyme parameter was set for 2 possible missed cleavages. The carbamidomethylation on cysteines was set a fixed modification, methionine oxidation, and N-terminal acetylation as the variable modifications. The results were filtered by a false discovery rate (FDR) of 1% on both precursor and protein level (Q value <0.01). RStudio (v4.2.3) was used for data analysis and visualization. Proteins were considered differentially expressed across different comparisons if absolute median ratio of the 2 conditions (*UNC13A* RNA versus a negative control RNA) was greater than 2 with an adjusted *P* value < 0.01 (Benjamini–Hochberg adjusted *P* value of a *t* test). Functional enrichment analysis was performed with clusterProfiler enrichGO function to identify GO categories by their biological processes (BP), molecular functions (MF), or cellular components (CC); and KEGG pathways. The signaling pathways with qi-value < 0.05 were considered significantly enriched.

### Other statistical analysis

Statistical information for each experiment, including the total number of samples and experiments analyzed and the specific tests performed, is reported in the figure legends. In general, data are presented as mean ± standard error of mean (SEM) and analyzed with one-way or two-way ANOVA followed by Tukey’s or Bonferroni’s post hoc analysis, unpaired Student’s *t* tests or Pearson’s correlation tests (GraphPad Prism, version 9.2.0). All data with *P* < 0.05 were considered statistically significant.

## Supporting information

S1 Fig TDP-43 can efficiently inhibit *UNC13A* cryptic exon inclusion independently of GWAS SNP. Related to Fig 1. (A) Schematic representation of GFP-tagged constructs for overexpressing wild-type TDP-43 (GFP-TDP-43_WT_) or an RNA-binding deficient TDP-43 mutant (GFP-TDP-43_5FL_). (B) qRT-PCR of *UNC13A* cryptic RNA confirmed that overexpression of GFP-TDP-43_WT_, but not GFP-TDP-43_5FL_, rescues *UNC13A* cryptic splicing in *TARDBP* KO HeLa cells. (C) qRT-PCR of *TARDBP* RNA confirmed similar expression of GFP-TDP-43_WT_ and GFP-TDP-43_5FL_. Graphs represent mean ± SEM of 3 independent replicates. Statistical differences were assessed by two-way ANOVA followed by Tukey’s multiple comparisons test (ns: not significant, *P < 0.05, **P < 0.005, ***P < 0.0005, ****P < 0.0001). Data used to generate the graphs in B and C can be found in **S3 Table**.
(PDF)

S2 Fig Pathway analyses of *UNC13A* RNA binders identified by proteomics reveal proteins involved in RNA metabolism. Related to Fig 3. Significant Gene Ontology terms (**A–C**) and KEGG pathways (**D**) are shown. Data used to generate the graphs in A–D can be found in **S2 Table**.
(PDF)

S3 Fig The expression levels of hnRNP L, hnRNP A1, and hnRNP A2B1 are not affected by TDP-43 depletion. Related to Fig 3. **(A)** Immunoblots of lysates from WT and *TARDBP* KO HeLa cells using antibodies against hnRNP L, hnRNP A1, hnRNP A2B1, TDP-43, and GAPDH were used as a loading control. Blots provided in Supporting information (**S1 Raw images**). (**B**) Densitometric analysis of the immunoblots showed comparable expression levels of hnRNP L, hnRNP A1, and hnRNP A2B1 between cells with (WT) and without (KO) TDP-43. Graphs represent mean ± SEM from 3 experimental replicates. Statistical differences were assessed by Student’s *t* test (ns: not significant, ***P < 0.001). Data used to generate the graphs in B can be found in **S3 Table**.
(PDF)

S4 Fig Endogenous hnRNP L bind *UNC13A* WT minigene RNA in *TARDBP* KO HeLa cells. Related to Fig 4. *TARDBP* KO HeLa cells overexpressing the *UNC13A* WT minigene were UV-crosslinked and hnRNP L-bound RNA was immunoprecipitated using a mouse monoclonal hnRNP L antibody [4D11] (ab6106, Abcam), as explained in Materials and methods. GFP immunoprecipitation served as negative control in the assay. qRT-PCR analysis demonstrates *UNC13A* RNA bound to endogenous hnRNP L but not GFP. Graph represents mean ± SEM of 3 independent replicates. Statistical differences were assessed by Student’s *t* test (***P < 0.0005). Data used to generate the graph can be found in **S3 Table**.
(PDF)

S5 Fig RNA-binding sites for hnRNP L, hnRNP A1, and hnRNP A2B1 were found in the intronic regions flanking the *UNC13A* cryptic exon. Related to Fig 4. UN*C13A* cryptic exon (chr19:17,753,223–17,753,350, hg19) and cryptic exon with flanking intronic (chr19:17,752,366–17,753,653, hg19) sequences were queried in a database containing known RNA-binding motifs (http://rbpmap.technion.ac.il/) to identify sequences within *UNC13A* where hnRNP L, hnRNP A1, and hnRNP A2B1 may bind. High stringency level settings were applied in which 2 thresholds are established: *p* value < 0.005 (significant hits) and *p* value <0.01 (suboptimal). Note the GWAS SNP located within the cryptic exon (chr19:17,753,239; hg19) is indicated in **A**. Results in B are the same in A but after also applying the conservation filter option, which uses UCSC phyloP conservation of placental mammals. This additional filter is recommended to increase specificity of results.
(PDF)

S6 Fig The deletion of *UNC13A* cryptic exon affects its binding ability to hnRNP L. Related to Fig 4. In vitro-transcribed RNA from WT and ΔCE *UNC13A* minigenes (**A**) were incubated with nuclear extracts from WT HeLa cells to assess their ability to bind the following proteins by western blot analyses after pull-down by hnRNP L (**B**). Blot provided in Supporting information (S1 Raw images). The graph shows reduced binding to ΔCE minigene by hnRNP L, as quantified by the signal intensity of the western blots using Image J. Graph represents mean ± SEM of 3 independent assays. Statistical differences were assessed by Student’s *t* test, *P < 0.05. Data used to generate the graph can be found in **S3 Table**.
(PDF)

S7 Fig Reducing levels of hnRNP L, hnRNPA1, or A2B1 under normal levels of TDP-43 does not lead to *UNC13A* cryptic exon inclusion. Related to Fig 4. WT *UNC13A* minigene was expressed in WT HeLa cells treated with either control (siControl) or siRNAs against *TARDBP* (siTARDBP), *HNRNPL* (siHNRPL), *HNRNPA1* (siHNRPA1), or *HNRNPA2B1* (siHNRNPA2B1). RNA was extracted, and qRT-PCR was performed to assess the expression levels of *UNC13A* cryptic (**Fig 4**), *TARDBP* (**A**), *HNRNPL* (**B**), *HNRNPA1* (**C**), or *HNRNPA2B1* (**D**) RNA. All graphs represent mean ± SEM from 3 independent experiments. Statistical differences were assessed by one-way ANOVA followed by Bonferroni’s multiple comparisons test (ns: not significant, **P < 0.005, ***P < 0.0005, ****P < 0.0001). Data used to generate the graphs in A–D can be found in S3 Table.
(PDF)

S8 Fig Down-regulation of *HNRNPL* further enhances *UNC13A* cryptic RNA containing the reference haplotype, in the context of *TARDBP* KO HeLa cells. Related to Fig 4. WT or CE SNP *UNC13A* minigenes were expressed in *TARDBP* KO HeLa cells treated with either control (siControl) or siRNAs against *HNRNPL* (siHNRPL) or *HNRNPA2B1* (siHNRNPA2B1), and RT-qPCR was performed to assess the expression levels of *UNC13A* cryptic (**A**), *HNRNPL* (**B**), or *HNRNPA2B1* (**C**) RNA. Statistical differences were assessed by two-way ANOVA followed by Bonferroni’s multiple comparisons test (ns: not significant, *P < 0.05, ****P < 0.0001). Data used to generate the graphs in A–C can be found in **S3 Table**.
(PDF)

S9 Fig hnRNP A1 and hnRNP A2B1 protein levels do not associate with *UNC13A cryptic* RNA levels. **Related to Fig 5A.** hnRNP A1 and hnRNP A2B1 protein levels were measured in frontal cortex samples from 54 FTLD-TDP cases by western blot and quantified by Image J. The associations of hnRNP A1 or hnRNP A2B1 protein levels with *UNC13A cryptic* RNA using Pearson correlation test are shown. Data used to generate the graphs in A and B can be found in **S3 Table**.
(PDF)

S1 Table Data used to generate the volcano plot in Fig 3C. (XLSX)

S2 Table Data used to generate graphs in S2 Fig. (XLSX)

S3 Table Data used to generate graphs in Figs 1, 2, 4–6, S1, S3, S4, and S6–S9. (XLSX)

S1 Raw images Uncropped blots from Figs 1B, 2B, 3B, 3D, 4, 5B, S3A, and S6B. (PDF)

## Abbreviations

ALS: amyotrophic lateral sclerosis
BP: biological process
CC: cellular component
CE: cryptic exon
CFTR: cystic fibrosis transmembrane conductance regulator
CLIP: cross-linking and immunoprecipitation
DIA: data independent acquisition
FDR: false discovery rate
FTD: frontotemporal dementia
GO: Gene Ontology
GWAS: genome-wide association study
hnRNP: heterogeneous nuclear ribonucleoprotein
IRB: Institutional Review Board
LC-MS: liquid chromatography–mass spectrometry
MF: molecular function
qRT-PCR: quantitative reverse transcription polymerase chain reaction
RBP: RNA-binding protein
SEM: standard error of mean
siRNA: small interfering RNA
SNP: single-nucleotide polymorphism
TDP-43: TAR DNA-binding protein-43
WT: wild-type
