# Correction: TDP-43 and other hnRNPs regulate cryptic exon inclusion of a key ALS/FTD risk gene, *UNC13A*

**DOI:** 10.1371/journal.pbio.3002228

**Published:** 2023-07-14

**Authors:** Yuka Koike, Sarah Pickles, Virginia Estades Ayuso, Karen Jansen-West, Yue A. Qi, Ziyi Li, Lillian M. Daughrity, Mei Yue, Yong-Jie Zhang, Casey N. Cook, Dennis W. Dickson, Michael Ward, Leonard Petrucelli, Mercedes Prudencio

In the Introduction, there is an error in the second sentence of the second paragraph. The correct sentence is: Cryptic exons contain parts of introns that are erroneously spliced into the mature mRNA.
