# Supplementary material for: TDP-43 and other hnRNPs regulate cryptic exon inclusion of a key ALS/FTD risk gene, UNC13A
Source: PLoS Biol. 2023 Mar 17;21(3):e3002028. doi: 10.1371/journal.pbio.3002028 (PMC10057836; doi:10.1371/journal.pbio.3002028)
Supplement: S1 Fig — Related to Fig 1. (A) Schematic representation of GFP-tagged constructs for overexpressing wild-type TDP-43 (GFP-TDP-43WT) or an RNA-binding deficient TDP-43 mutant (GFP-TDP-435FL). (B) qRT-PCR of UNC13A cryptic RNA confirmed that overexpression of GFP-TDP-43WT, but not GFP-TDP-435FL, rescues UNC13A cryptic splicing in TARDBP KO HeLa cells. (C) qRT-PCR of TARDBP RNA confirmed similar expression of GFP-TDP-43WT and GFP-TDP-435FL. Graphs represent mean ± SEM of 3 independent replicates. Statistical differences were assessed by two-way ANOVA followed by Tukey’s multiple comparisons test (ns: not significant, *P < 0.05, **P < 0.005, ***P < 0.0005, ****P < 0.0001). Data used to generate the graphs in B and C can be found in S3 Table. (PDF) [file pbio.3002028.s001.pdf]

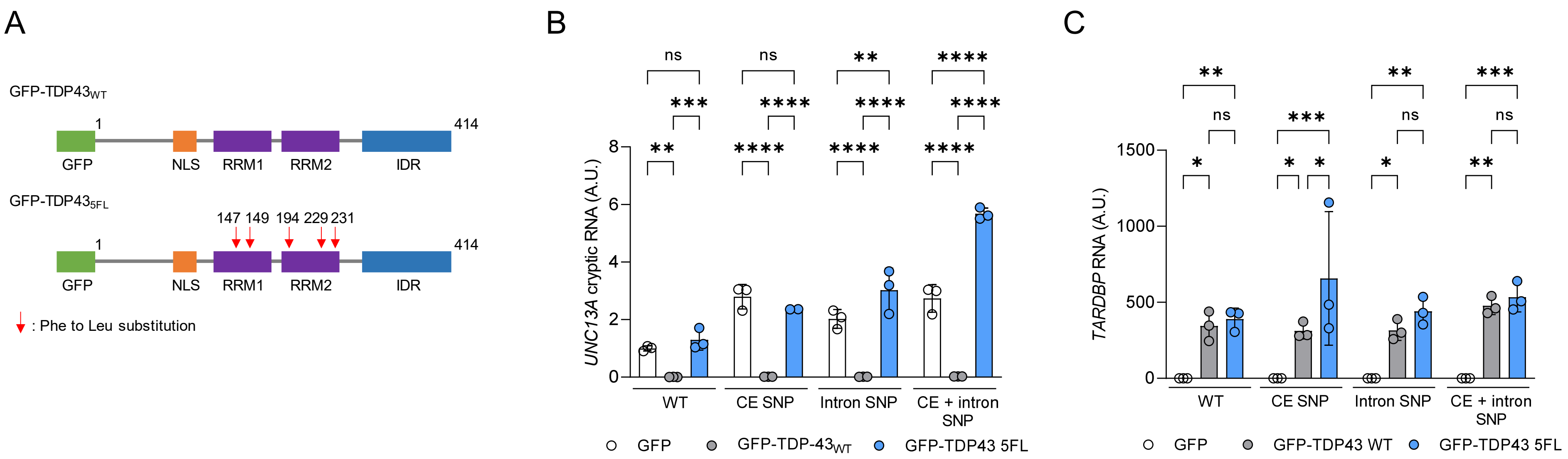

S1 Fig. TDP-43 can efficiently inhibit *UNC13A* cryptic exon inclusion independently of GWAS SNP. Related to Fig 1. (**A**) Schematic representation of GFP-tagged constructs for overexpressing wild type TDP-43 (GFP-TDP-43_{WT}) or an RNA-binding deficient TDP-43 mutant (GFP-TDP-43_{5FL}). (**B**) qRT-PCR of *UNC13A* cryptic RNA confirmed that overexpression of GFP-TDP-43_{WT}, but not GFP-TDP-43_{5FL}, rescues *UNC13A* cryptic splicing in *TARDBP* KO HeLa cells. (**C**) qRT-PCR of *TARDBP* RNA confirmed similar expression of GFP-TDP-43_{WT} and GFP-TDP-43_{5FL}. Graphs represent mean ± s.e.m. of three independent replicates. Statistical differences were assessed by Two-way ANOVA followed by Tukey's multiple comparisons test (ns: not significant, *P < 0.05, **P < 0.005, ***P < 0.0005, ****P <0.0001). Data used to generate the graphs in B-C can be found in **S3 Table**.
