# Supplementary material for: TDP-43 and other hnRNPs regulate cryptic exon inclusion of a key ALS/FTD risk gene, UNC13A
Source: PLoS Biol. 2023 Mar 17;21(3):e3002028. doi: 10.1371/journal.pbio.3002028 (PMC10057836; doi:10.1371/journal.pbio.3002028)
Supplement: S2 Fig — Related to Fig 3. Significant Gene Ontology terms (A–C) and KEGG pathways (D) are shown. Data used to generate the graphs in A–D can be found in S2 Table. (PDF) [file pbio.3002028.s002.pdf]

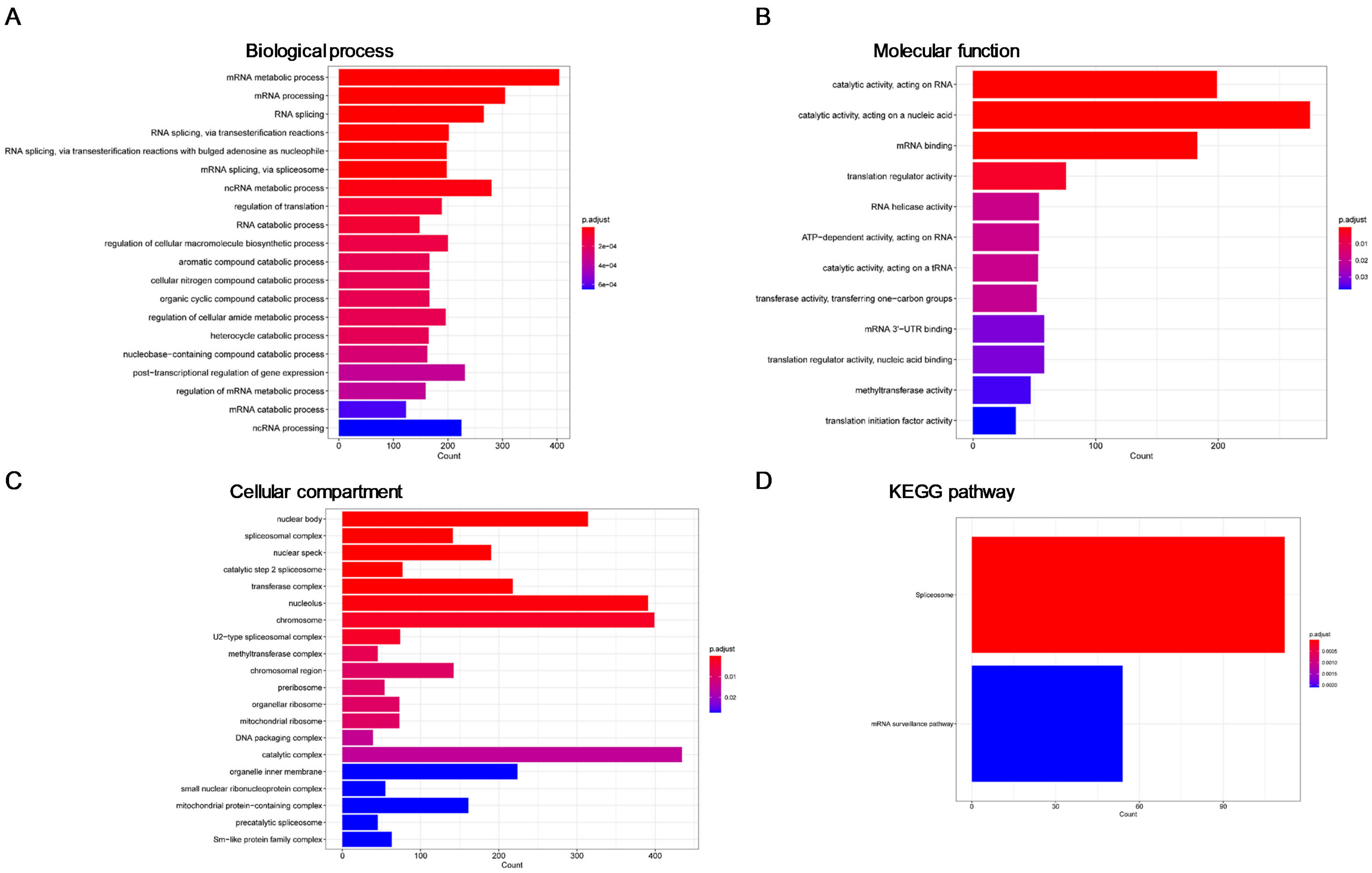

S2 Fig. Pathway analyses of *UNC13A* RNA binders identified by proteomics reveal proteins involved in RNA metabolism. Related to Fig 3. Significant Gene Ontology terms (**A-C**) and KEGG pathways (**D**) are shown. Data used to generate the graphs in A-D can be found in **S2 Table**.
