# Supplementary material for: TDP-43 and other hnRNPs regulate cryptic exon inclusion of a key ALS/FTD risk gene, UNC13A
Source: PLoS Biol. 2023 Mar 17;21(3):e3002028. doi: 10.1371/journal.pbio.3002028 (PMC10057836; doi:10.1371/journal.pbio.3002028)
Supplement: S3 Fig — Related to Fig 3. (A) Immunoblots of lysates from WT and TARDBP KO HeLa cells using antibodies against hnRNP L, hnRNP A1, hnRNP A2B1, TDP-43, and GAPDH were used as a loading control. Blots provided in Supporting information (S1 Raw images). (B) Densitometric analysis of the immunoblots showed comparable expression levels of hnRNP L, hnRNP A1, and hnRNP A2B1 between cells with (WT) and without (KO) TDP-43. Graphs represent mean ± SEM from 3 experimental replicates. Statistical differences were assessed by Student’s t test (ns: not significant, ***P < 0.001). Data used to generate the graphs in B can be found in S3 Table. (PDF) [file pbio.3002028.s003.pdf]

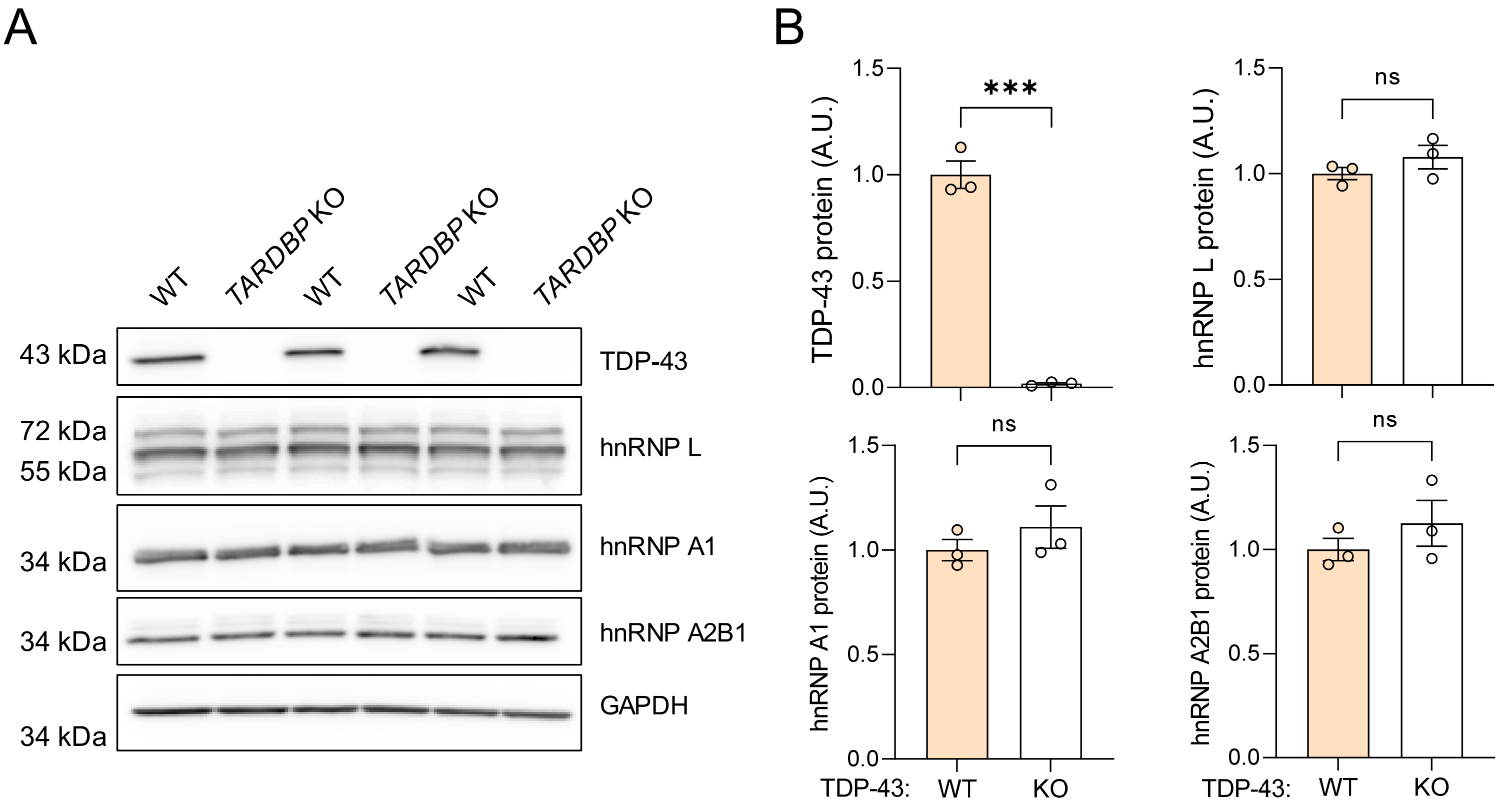

S3 Fig. The expression levels of hnRNP L, hnRNP A1 and hnRNP A2B1 are not affected by TDP-43 depletion. Related to Fig 3. (A) Immunoblots of lysates from WT and *TARDBP* KO HeLa cells using antibodies against hnRNP L, hnRNP A1, hnRNP A2B1, TDP-43, and GAPDH was used as a loading control. Blots provided in Supporting information (**S1 Raw Images**). (**B**) Densitometric analysis of the immunoblots showed comparable expression levels of hnRNP L, hnRNP A1 and hnRNP A2B1 between cells with (WT) and without (KO) TDP-43. Graphs represent mean ± s.e.m. from three experimental replicates. Statistical differences were assessed by Student's *t* test (ns: not significant, ***$P < 0.001$). Data used to generate the graphs in B can be found in **S3 Table**.
