# Supplementary material for: TDP-43 and other hnRNPs regulate cryptic exon inclusion of a key ALS/FTD risk gene, UNC13A
Source: PLoS Biol. 2023 Mar 17;21(3):e3002028. doi: 10.1371/journal.pbio.3002028 (PMC10057836; doi:10.1371/journal.pbio.3002028)
Supplement: S4 Fig — Related to Fig 4. TARDBP KO HeLa cells overexpressing the UNC13A WT minigene were UV-crosslinked and hnRNP L-bound RNA was immunoprecipitated using a mouse monoclonal hnRNP L antibody [4D11] (ab6106, Abcam), as explained in Materials and methods. GFP immunoprecipitation served as negative control in the assay. qRT-PCR analysis demonstrates UNC13A RNA bound to endogenous hnRNP L but not GFP. Graph represents mean ± SEM of 3 independent replicates. Statistical differences were assessed by Student’s t test (***P < 0.0005). Data used to generate the graph can be found in S3 Table. (PDF) [file pbio.3002028.s004.pdf]

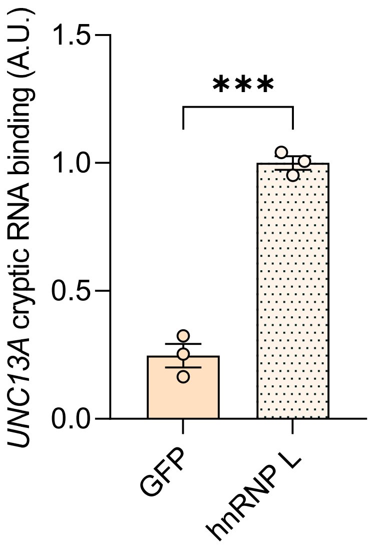

S4 Fig. Endogenous hnRNP L bind *UNC13A* WT minigene RNA in *TARDBP* KO HeLa cells. Related to Fig 4. *TARDBP* KO HeLa cells overexpressing the *UNC13A* WT minigene were UV-crosslinked and hnRNP L-bound RNA was immunoprecipitated using a mouse monoclonal hnRNP L antibody [4D11] (ab6106, Abcam), as explained in Materials and Methods. GFP immunoprecipitation served as negative control in the assay. qRT-PCR analysis demonstrates *UNC13A* RNA bound to endogenous hnRNP L but not GFP. Graph represents mean ± s.e.m. of three independent replicates. Statistical differences were assessed by Student's t-test (***$P < 0.0005$). Data used to generate the graph can be found in **S3 Table**.
