# Supplementary material for: TDP-43 and other hnRNPs regulate cryptic exon inclusion of a key ALS/FTD risk gene, UNC13A
Source: PLoS Biol. 2023 Mar 17;21(3):e3002028. doi: 10.1371/journal.pbio.3002028 (PMC10057836; doi:10.1371/journal.pbio.3002028)
Supplement: S5 Fig — Related to Fig 4. UNC13A cryptic exon (chr19:17,753,223–17,753,350, hg19) and cryptic exon with flanking intronic (chr19:17,752,366–17,753,653, hg19) sequences were queried in a database containing known RNA-binding motifs (http://rbpmap.technion.ac.il/) to identify sequences within UNC13A where hnRNP L, hnRNP A1, and hnRNP A2B1 may bind. High stringency level settings were applied in which 2 thresholds are established: p value < 0.005 (significant hits) and p value <0.01 (suboptimal). Note the GWAS SNP located within the cryptic exon (chr19:17,753,239; hg19) is indicated in A. Results in B are the same in A but after also applying the conservation filter option, which uses UCSC phyloP conservation of placental mammals. This additional filter is recommended to increase specificity of results. (PDF) [file pbio.3002028.s005.pdf]

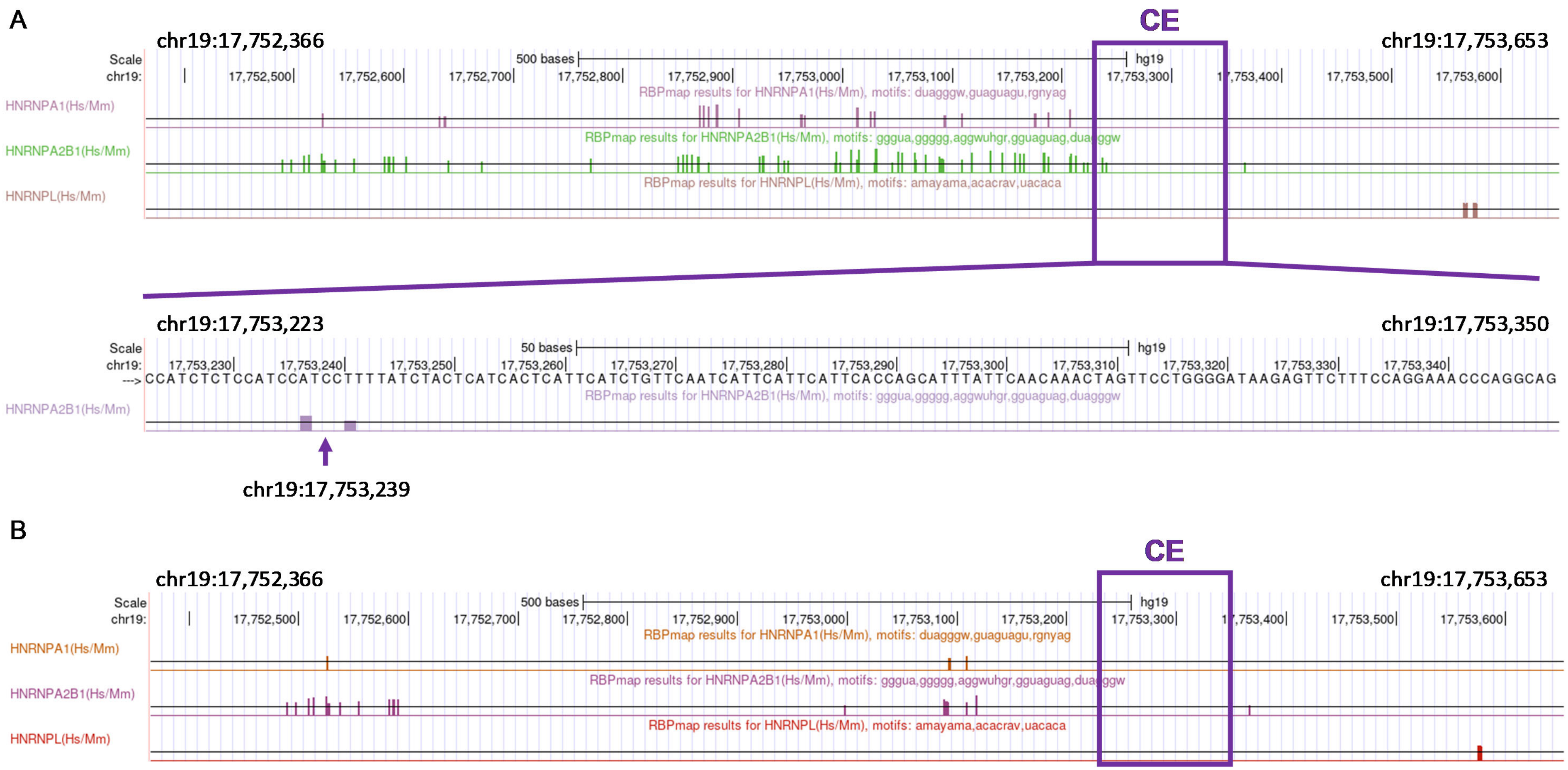

S5 Fig. RNA binding sites for hnRNP L, hnRNP A1 and hnRNP A2B1 were found in the intronic regions flanking the *UNC13A* cryptic exon. Related to Fig 4. *UNC13A* cryptic exon (chr19:17,753,223-17,753,350, hg19) and cryptic exon with flanking intronic (chr19:17,752,366-17,753,653, hg19) sequences were queried in a database containing known RNA binding motifs (http://rbpmap.technion.ac.il/) to identify sequences withing *UNC13A* where hnRNP L, hnRNP A1 and hnRNP A2B1 may bind. High stringency level settings were applied in which two thresholds are established: p value < 0.005 (significant hits) and p value <0.01 (suboptimal). Note the GWAS SNP located within the cryptic exon (chr19:17,753,239; hg19) is indicated in **A**. Results in B are the same in A but after also applying the conservation filter option, which uses UCSC phyloP conservation of placental mammals. This additional filter is recommended to increase specificity of results.
