# Supplementary material for: TDP-43 and other hnRNPs regulate cryptic exon inclusion of a key ALS/FTD risk gene, UNC13A
Source: PLoS Biol. 2023 Mar 17;21(3):e3002028. doi: 10.1371/journal.pbio.3002028 (PMC10057836; doi:10.1371/journal.pbio.3002028)
Supplement: S6 Fig — Related to Fig 4. In vitro-transcribed RNA from WT and ΔCE UNC13A minigenes (A) were incubated with nuclear extracts from WT HeLa cells to assess their ability to bind the following proteins by western blot analyses after pull-down by hnRNP L (B). Blot provided in Supporting information (S1 Raw images). The graph shows reduced binding to ΔCE minigene by hnRNP L, as quantified by the signal intensity of the western blots using Image J. Graph represents mean ± SEM of 3 independent assays. Statistical differences were assessed by Student’s t test, *P < 0.05. Data used to generate the graph can be found in S3 Table. (PDF) [file pbio.3002028.s006.pdf]

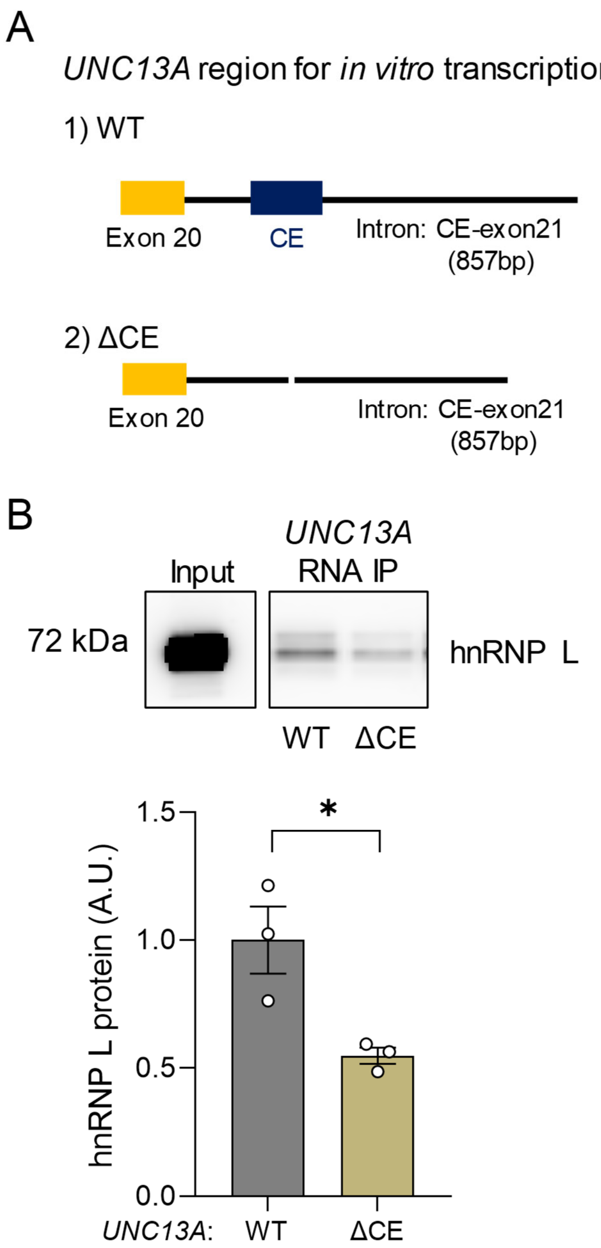

S6 Fig. The deletion of *UNC13A* cryptic exon affects its binding ability to hnRNP L. Related to Fig 4. *In vitro*-transcribed RNA from WT and ΔCE *UNC13A* minigenes (**A**) were incubated with nuclear extracts from WT HeLa cells to assess their ability to bind the following proteins by Western blot analyses after pull-down by hnRNP L (**B**). Blot provided in Supporting information (S1_raw_images). The graph shows reduced binding to ΔCE minigene by hnRNP L, as quantified by the signal intensity of the Western blots using Image J. Graph represents mean ± s.e.m. of three independent assays. Statistical differences were assessed by Student's *t*-test, *$P < 0.05$. Data used to generate the graph can be found in **S3 Table**.
