# Supplementary material for: TDP-43 and other hnRNPs regulate cryptic exon inclusion of a key ALS/FTD risk gene, UNC13A
Source: PLoS Biol. 2023 Mar 17;21(3):e3002028. doi: 10.1371/journal.pbio.3002028 (PMC10057836; doi:10.1371/journal.pbio.3002028)
Supplement: S7 Fig — Related to Fig 4. WT UNC13A minigene was expressed in WT HeLa cells treated with either control (siControl) or siRNAs against TARDBP (siTARDBP), HNRNPL (siHNRPL), HNRNPA1 (siHNRPA1), or HNRNPA2B1 (siHNRNPA2B1). RNA was extracted, and qRT-PCR was performed to assess the expression levels of UNC13A cryptic (Fig 4), TARDBP (A), HNRNPL (B), HNRNPA1 (C), or HNRNPA2B1 (D) RNA. All graphs represent mean ± SEM from 3 independent experiments. Statistical differences were assessed by one-way ANOVA followed by Bonferroni’s multiple comparisons test (ns: not significant, **P < 0.005, ***P < 0.0005, ****P < 0.0001). Data used to generate the graphs in A–D can be found in S3 Table. (PDF) [file pbio.3002028.s007.pdf]

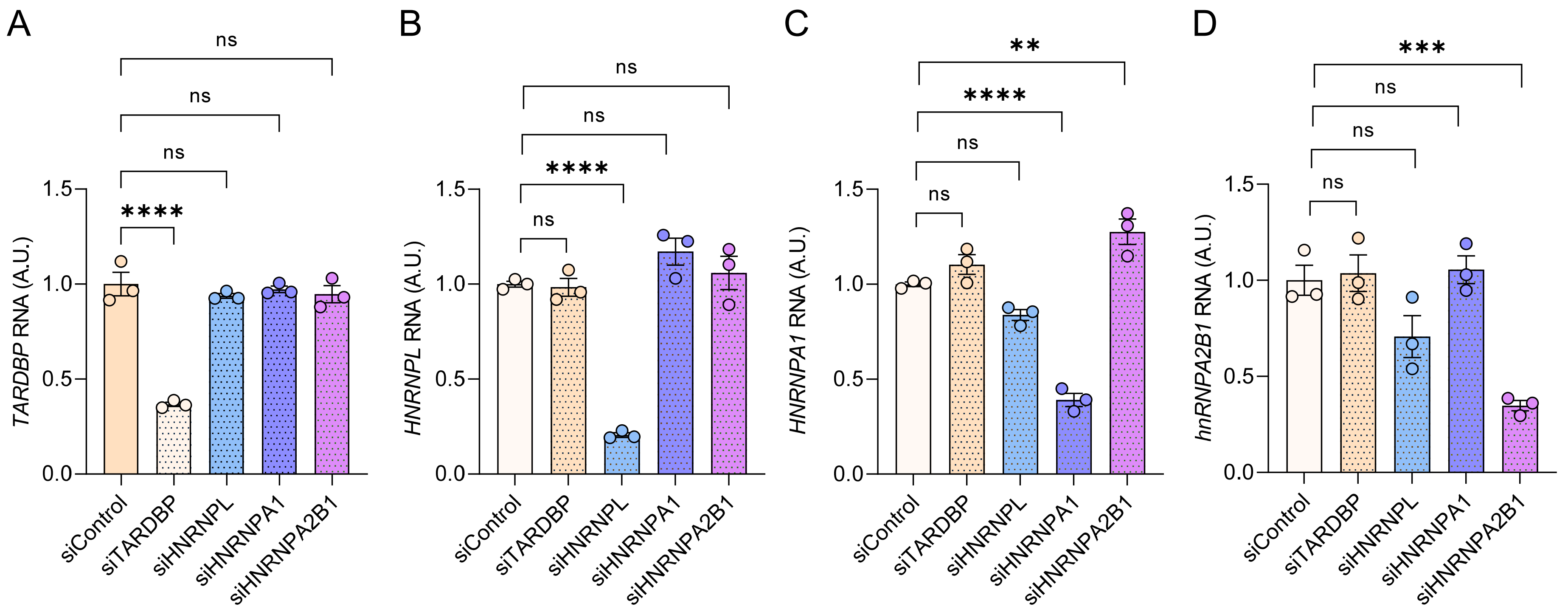

S7 Fig. Reducing levels of hnRNP L, hnRNPA1 or A2B1 under normal levels of TDP-43 does not lead to *UNC13A* cryptic exon inclusion. Related to Fig 4. WT *UNC13A* minigene was expressed in WT HeLa cells treated with either control (siControl) or siRNAs against *TARDBP* (siTARDBP), *HNRNPL* (siHNRPL), *HNRNPA1* (siHNRPA1) or *HNRNPA2B1* (siHNRNPA2B1). RNA was extracted, and qRT-PCR was performed to assess the expression levels of *UNC13A* cryptic (**Fig 4**), *TARDBP* (**A**), *HNRNPL* (**B**), *HNRNPA1* (**C**) or *HNRNPA2B1* (**D**) RNA. All graphs represent mean ± s.e.m. from three independent experiments. Statistical differences were assessed by One-way ANOVA followed by Bonferroni's multiple comparisons test (ns: not significant, **P < 0.005, ***P < 0.0005, ****P <0.0001). Data used to generate the graphs in A-D can be found in **S3 Table**.
