# Supplementary material for: TDP-43 and other hnRNPs regulate cryptic exon inclusion of a key ALS/FTD risk gene, UNC13A
Source: PLoS Biol. 2023 Mar 17;21(3):e3002028. doi: 10.1371/journal.pbio.3002028 (PMC10057836; doi:10.1371/journal.pbio.3002028)
Supplement: S8 Fig — Related to Fig 4. WT or CE SNP UNC13A minigenes were expressed in TARDBP KO HeLa cells treated with either control (siControl) or siRNAs against HNRNPL (siHNRPL) or HNRNPA2B1 (siHNRNPA2B1), and RT-qPCR was performed to assess the expression levels of UNC13A cryptic (A), HNRNPL (B), or HNRNPA2B1 (C) RNA. Statistical differences were assessed by two-way ANOVA followed by Bonferroni’s multiple comparisons test (ns: not significant, *P < 0.05, ****P < 0.0001). Data used to generate the graphs in A–C can be found in S3 Table. (PDF) [file pbio.3002028.s008.pdf]

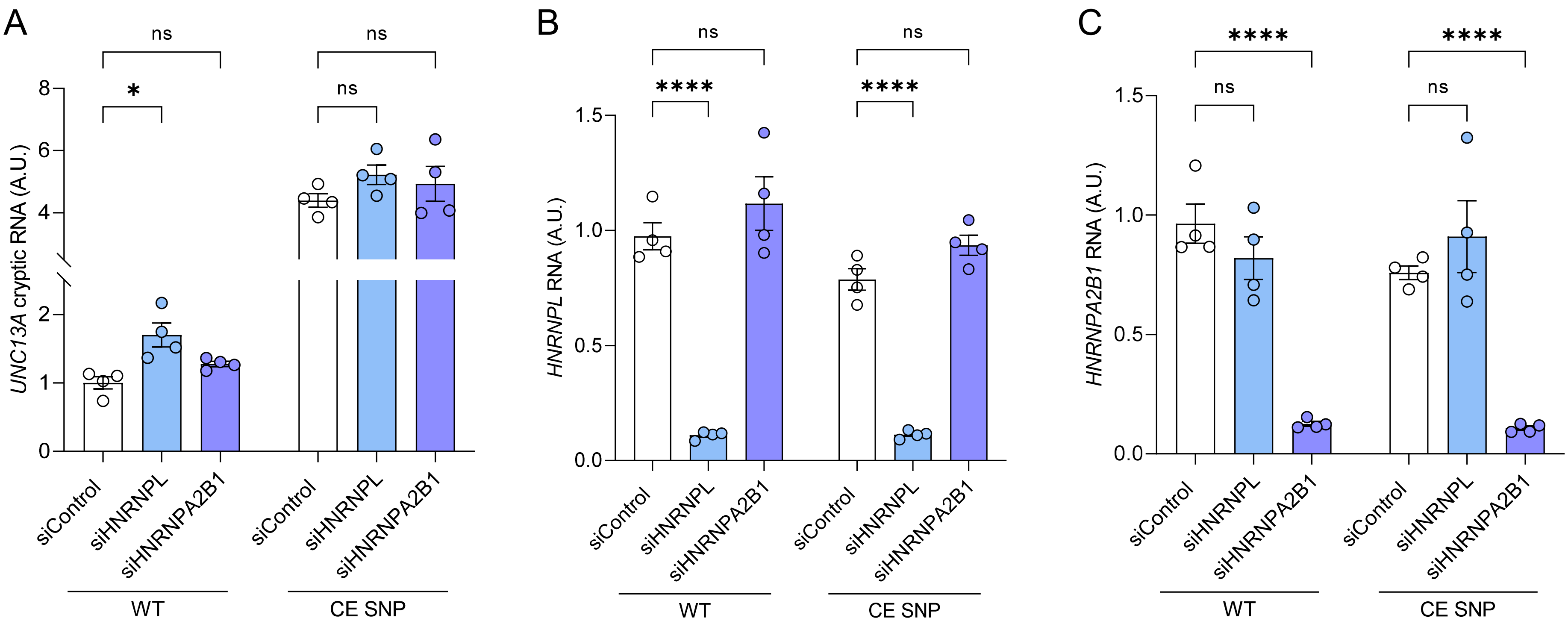

S8 Fig. Downregulation of *HNRNPL* further enhances *UNC13A* cryptic RNA containing the reference haplotype, in the context of *TARDBP* KO HeLa cells. Related to Fig 4. WT or CE SNP *UNC13A* minigenes were expressed in *TARDBP* KO HeLa cells treated with either control (siControl), or siRNAs against *HNRNPL* (siHNRPL) or *HNRNPA2B1* (siHNRNPA2B1), and RT-qPCR was performed to assess the expression levels of *UNC13A* cryptic (**A**), *HNRNPL* (**B**) or *HNRNPA2B1* (**C**) RNA. Statistical differences were assessed by Two-way ANOVA followed by Bonferroni's multiple comparisons test (ns: not significant, *P < 0.05, ****P <0.0001). Data used to generate the graphs in A-C can be found in **S3 Table**.
