# Supplementary material for: TDP-43 and other hnRNPs regulate cryptic exon inclusion of a key ALS/FTD risk gene, UNC13A
Source: PLoS Biol. 2023 Mar 17;21(3):e3002028. doi: 10.1371/journal.pbio.3002028 (PMC10057836; doi:10.1371/journal.pbio.3002028)
Supplement: S9 Fig — Related to Fig 5A. hnRNP A1 and hnRNP A2B1 protein levels were measured in frontal cortex samples from 54 FTLD-TDP cases by western blot and quantified by Image J. The associations of hnRNP A1 or hnRNP A2B1 protein levels with UNC13A cryptic RNA using Pearson correlation test are shown. Data used to generate the graphs in A and B can be found in S3 Table. (PDF) [file pbio.3002028.s009.pdf]

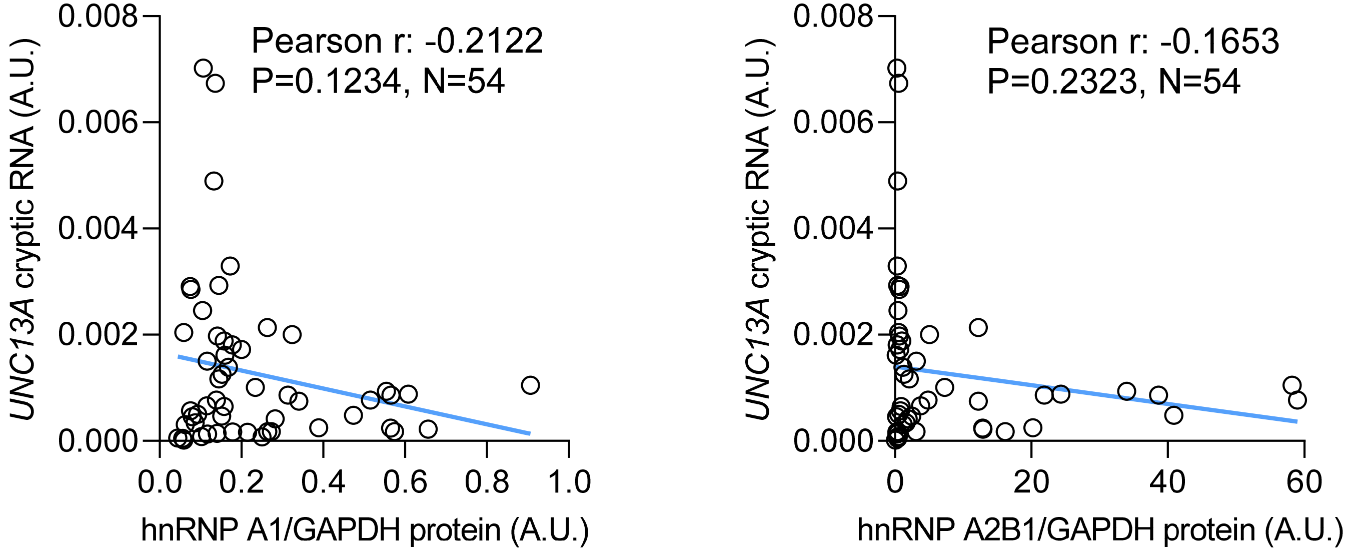

S9 Fig. hnRNP A1 and hnRNP A2B1 protein levels do not associate with *UNC13A cryptic* RNA levels. Related to Fig 5A. hnRNP A1 and hnRNP A2B1 protein levels were measured in frontal cortex samples from 54 FTLD-TDP cases by Western blot and quantified by Image J. The associations of hnRNP A1 or hnRNP A2B1 protein levels with *UNC13A cryptic* RNA using Pearson correlation test are shown. Data used to generate the graphs in A-B can be found in **S3 Table**.
