# Supplementary material for: TDP-43 and other hnRNPs regulate cryptic exon inclusion of a key ALS/FTD risk gene, UNC13A
Source: PLoS Biol. 2023 Mar 17;21(3):e3002028. doi: 10.1371/journal.pbio.3002028 (PMC10057836; doi:10.1371/journal.pbio.3002028)

Raw uncropped images for Figures 1B, 2B, 3B, 3D, 4A-D, 5B, S3A, S6B

Raw uncropped images for Figure 1B

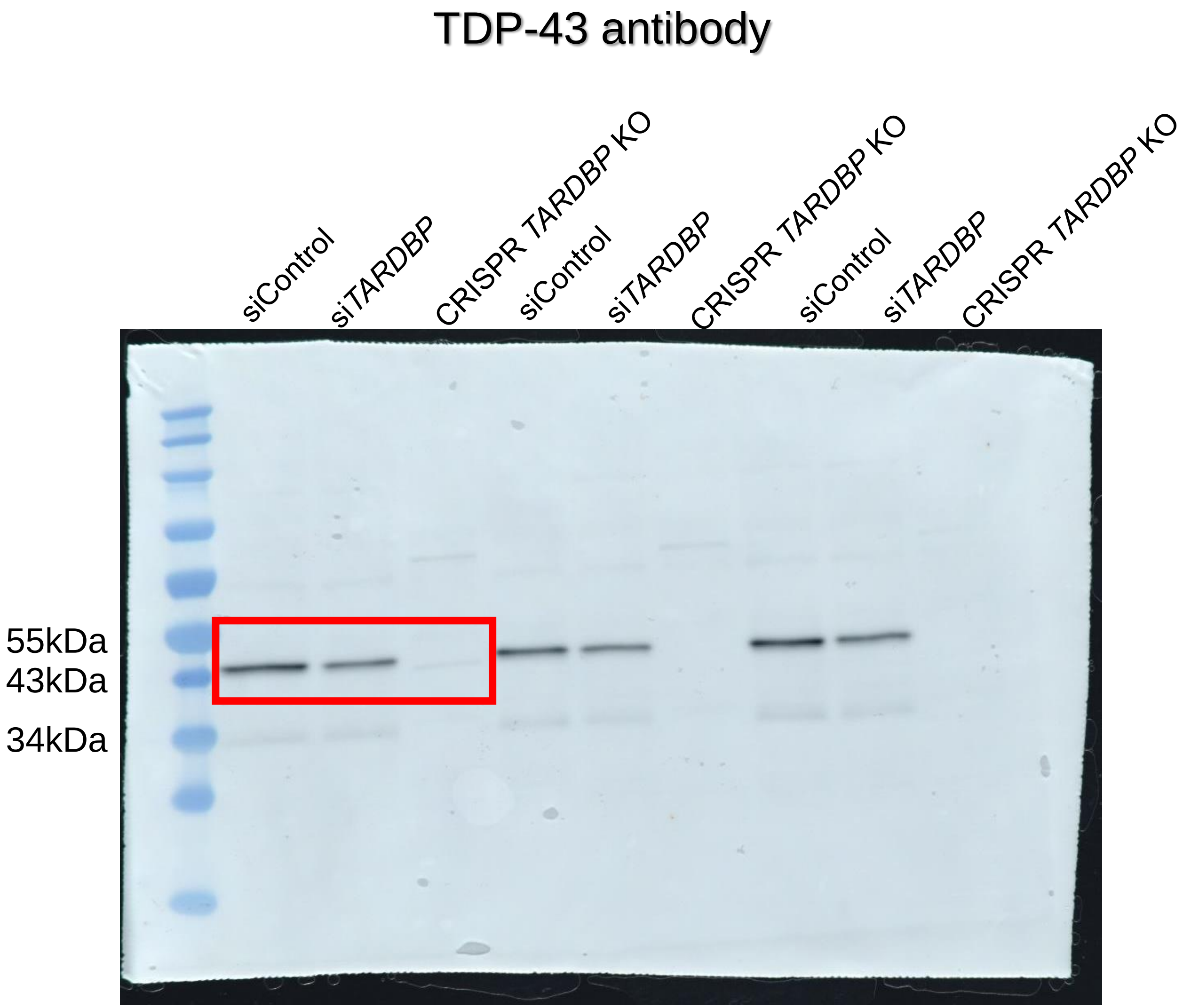

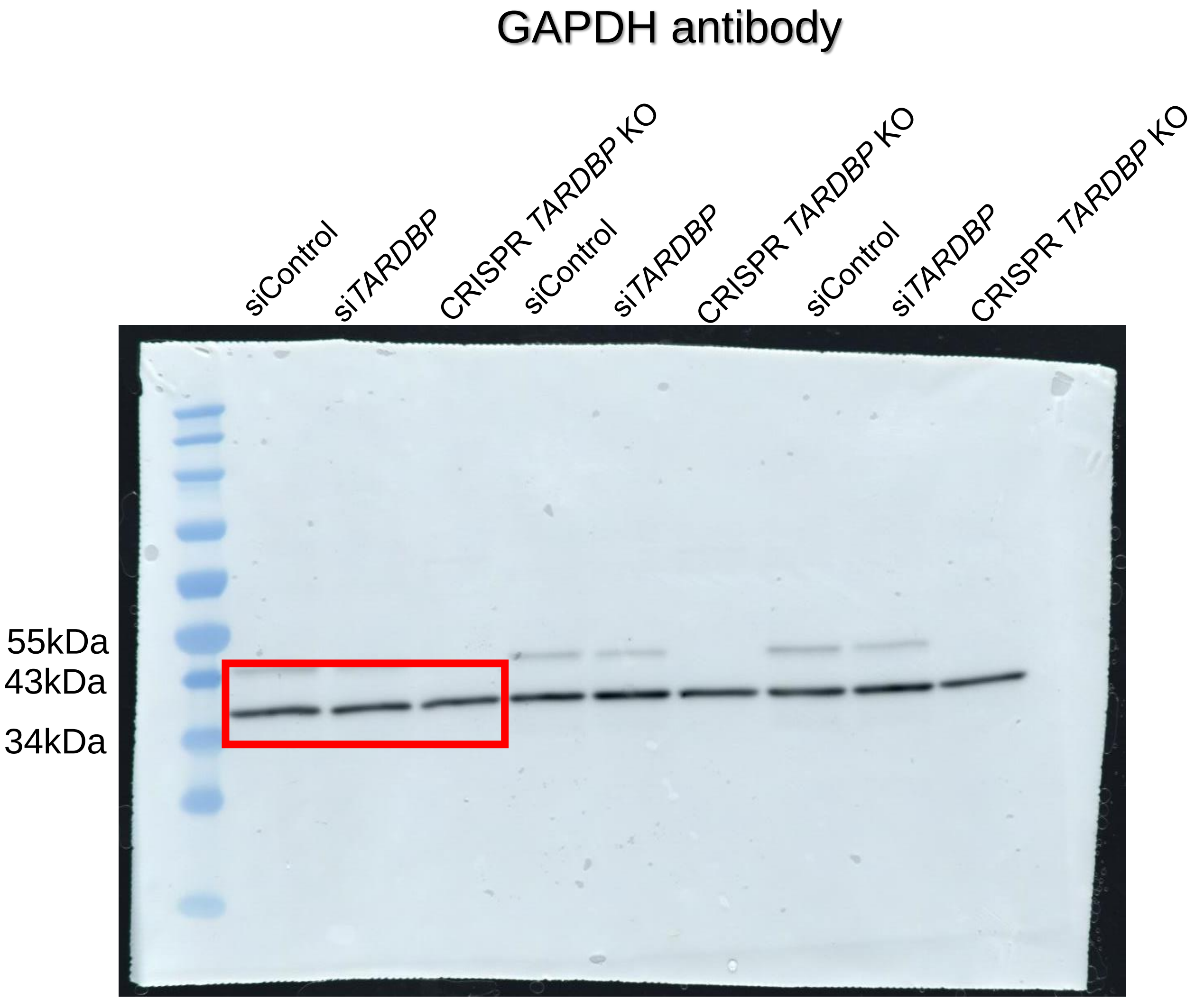

Raw uncropped images for Figure 2B

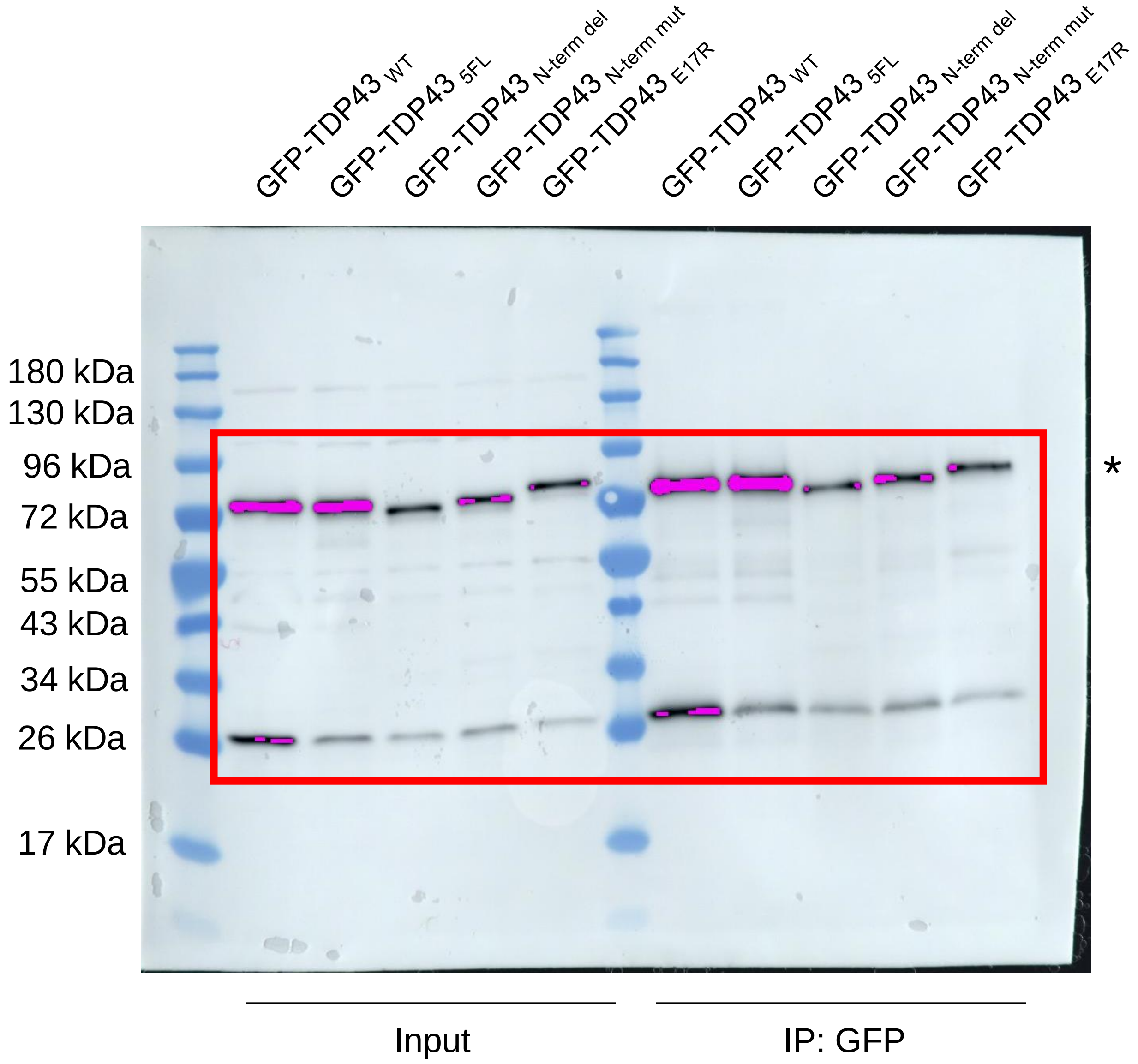

*GFP-TDP-43

Raw uncropped images for Figure 3B

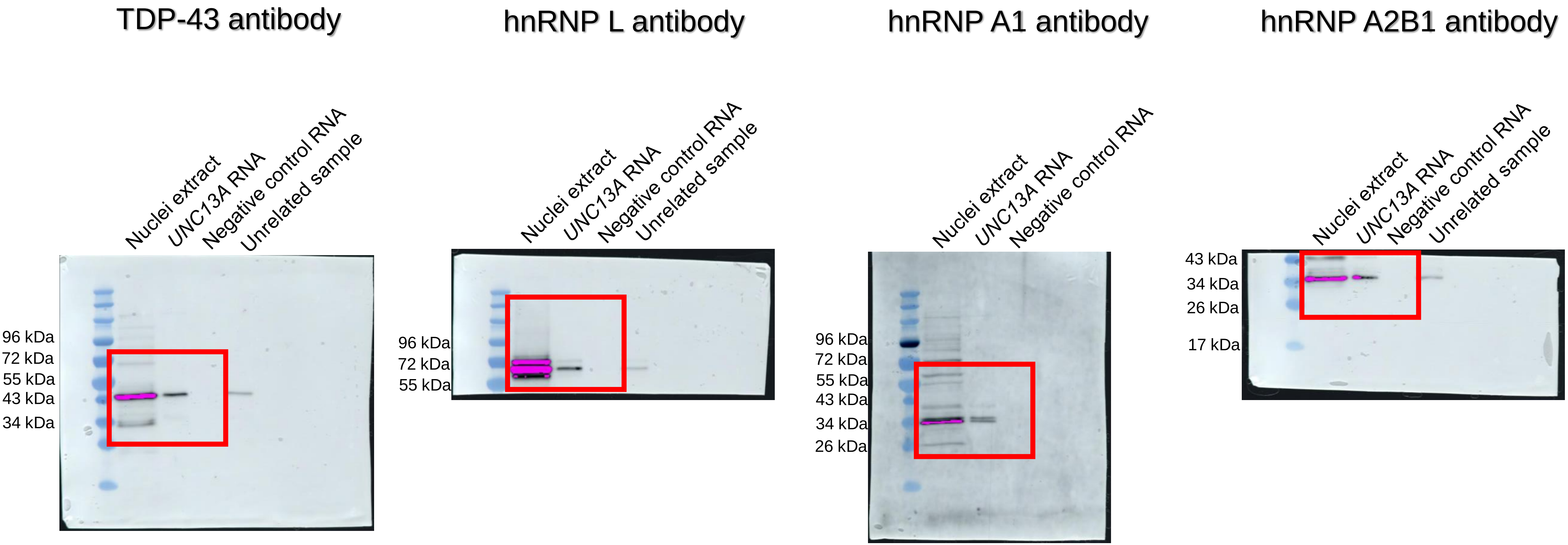

Raw uncropped images for Figure 3D

1. WT HeLa cells
2. *TARDBP* KO HeLa cells
3. *TARDBP* KO HeLa cells treated with siRNA against *HNRNPL*, *HNRNPA2B1* and *HNRNPA1*

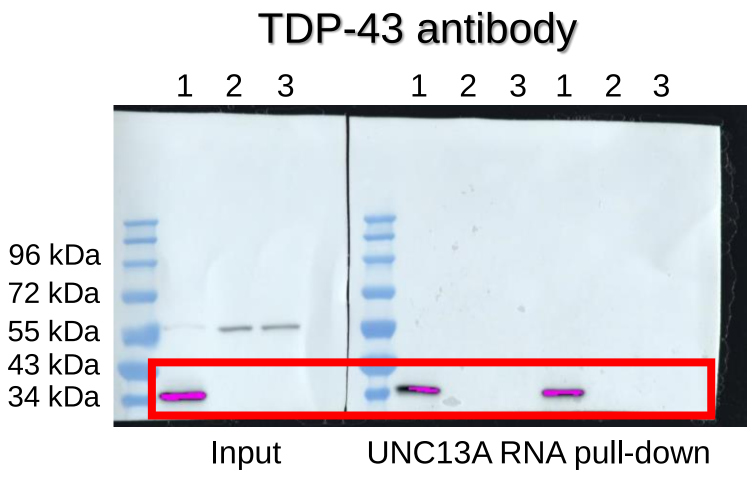

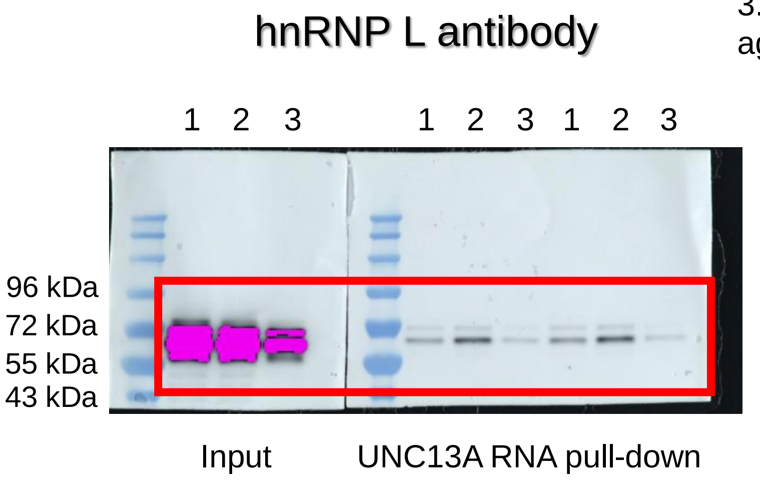

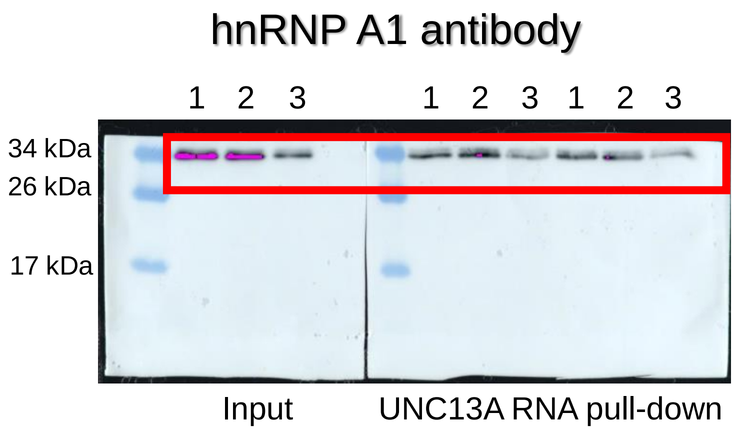

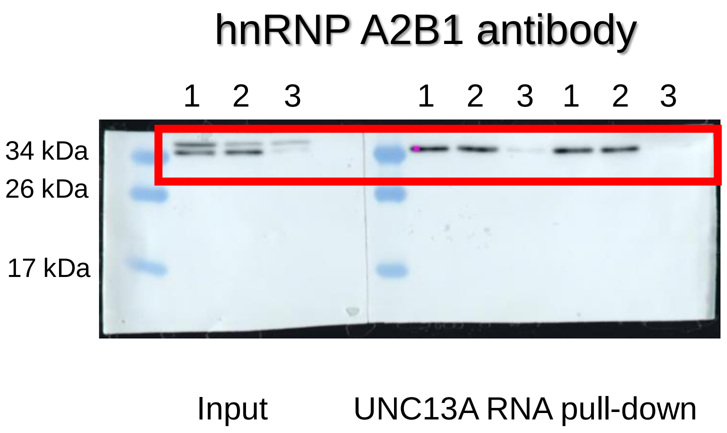

Raw uncropped images for Figure 4A-D

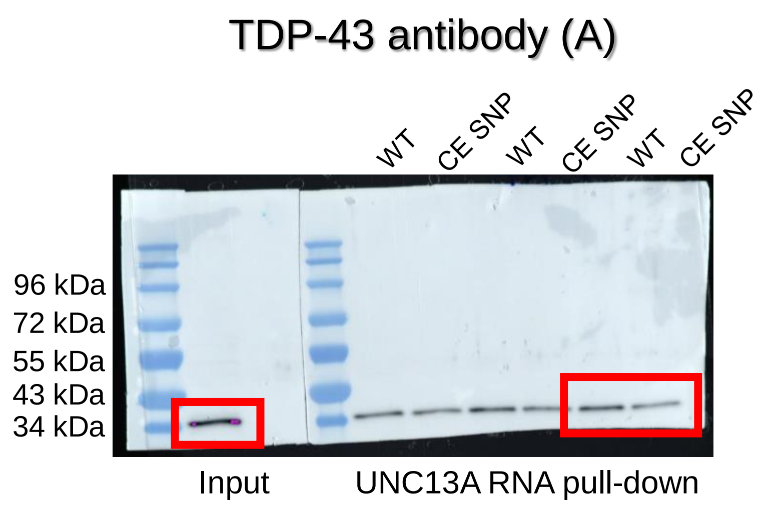

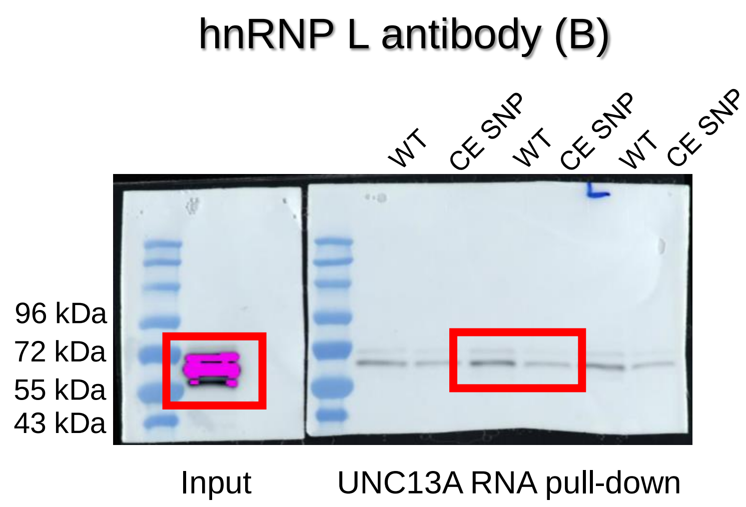

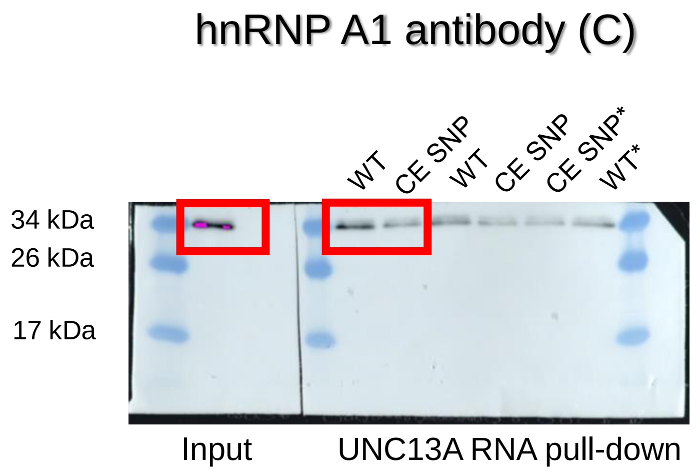

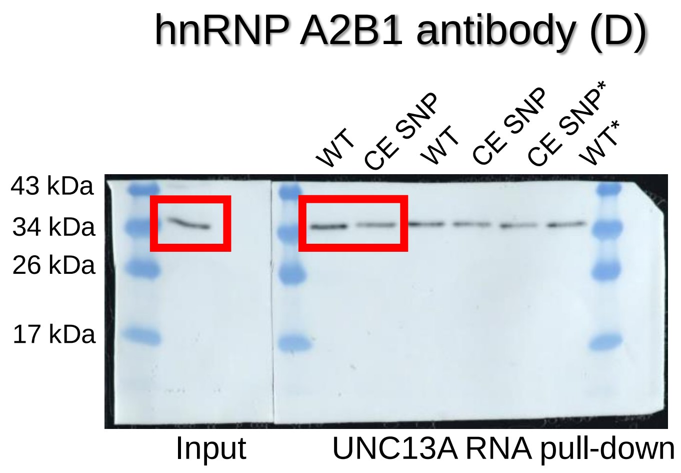

Raw uncropped images for Figure 5B

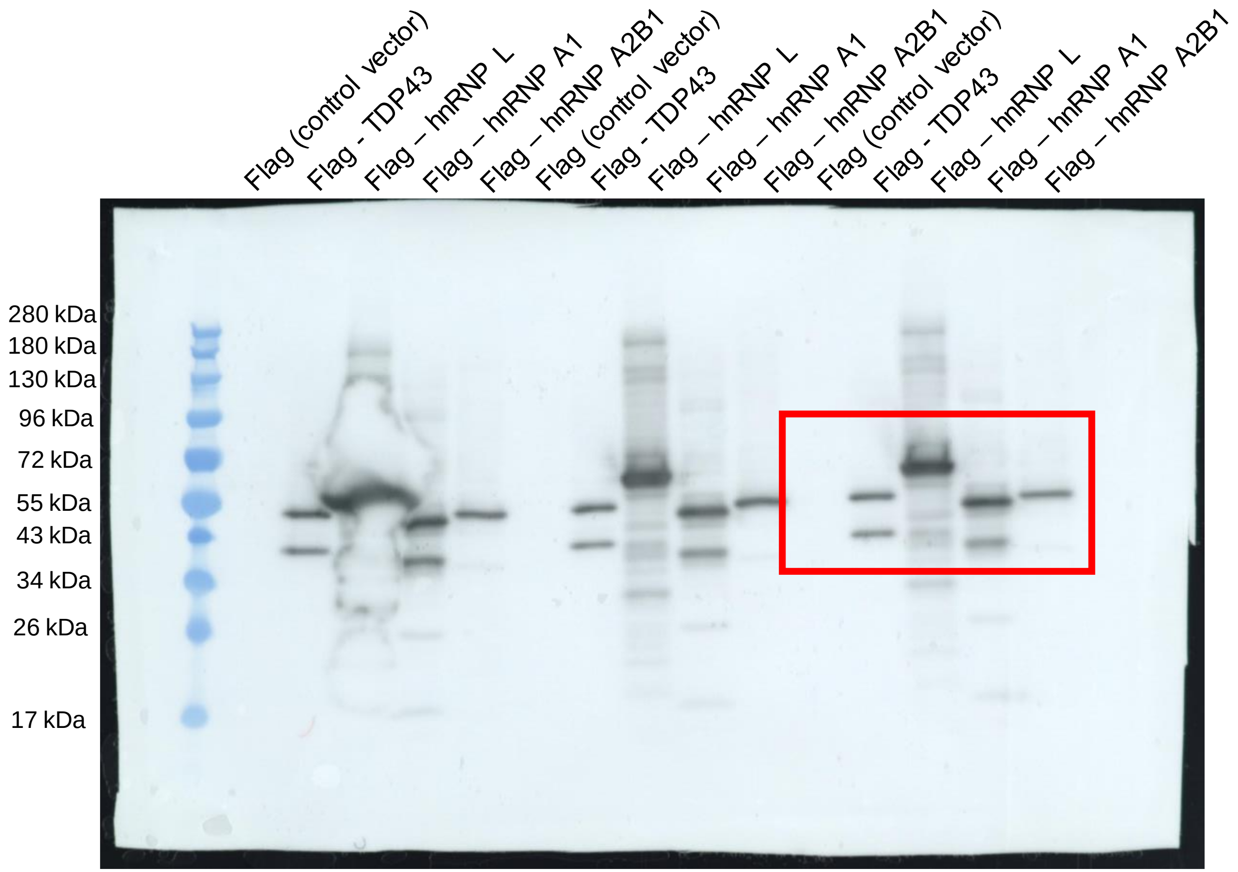

Raw uncropped images for Figure S3A

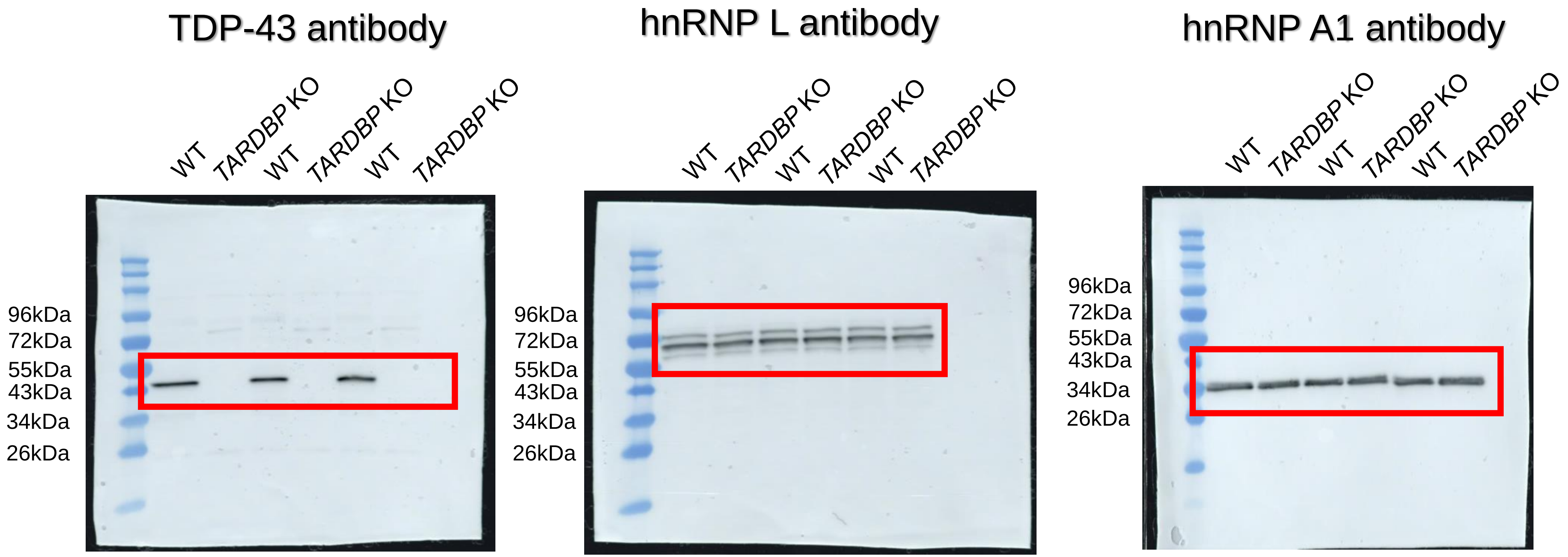

Raw uncropped images for Figure S3A

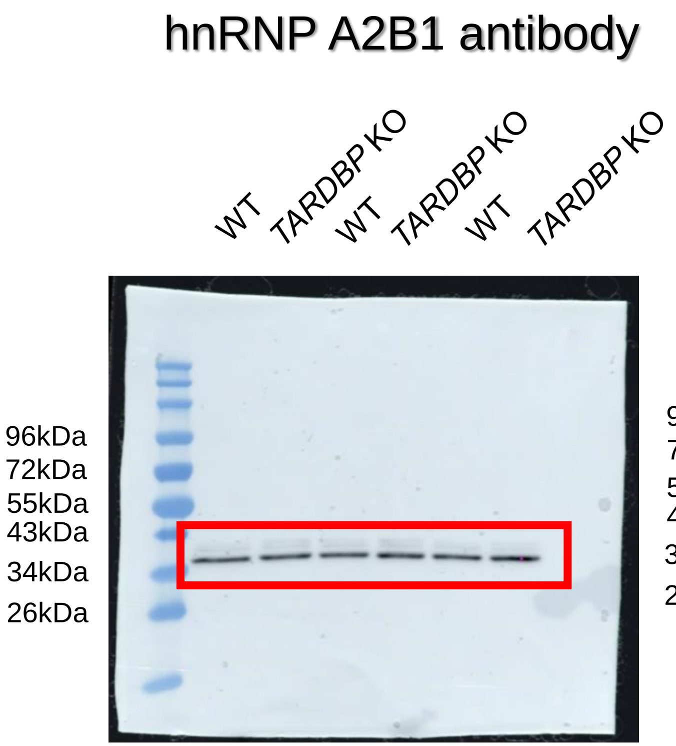

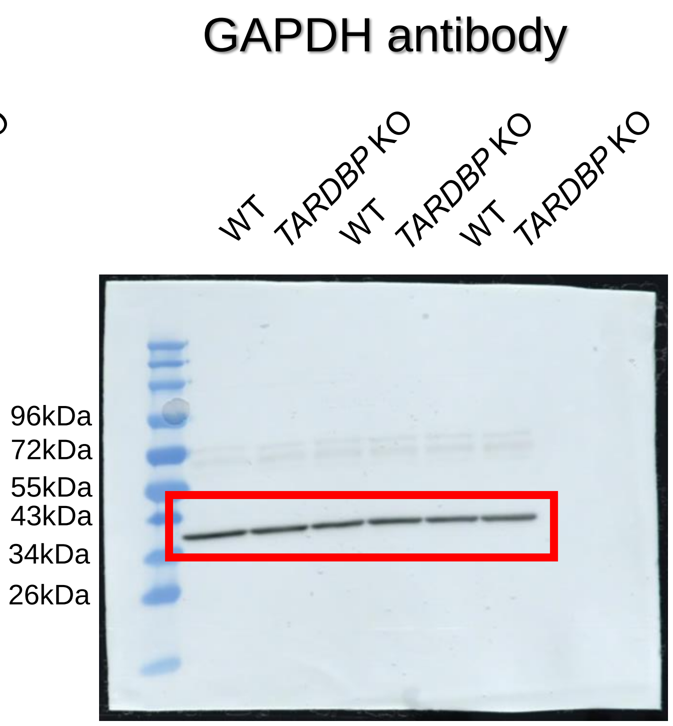

Raw uncropped images for Figure S6B

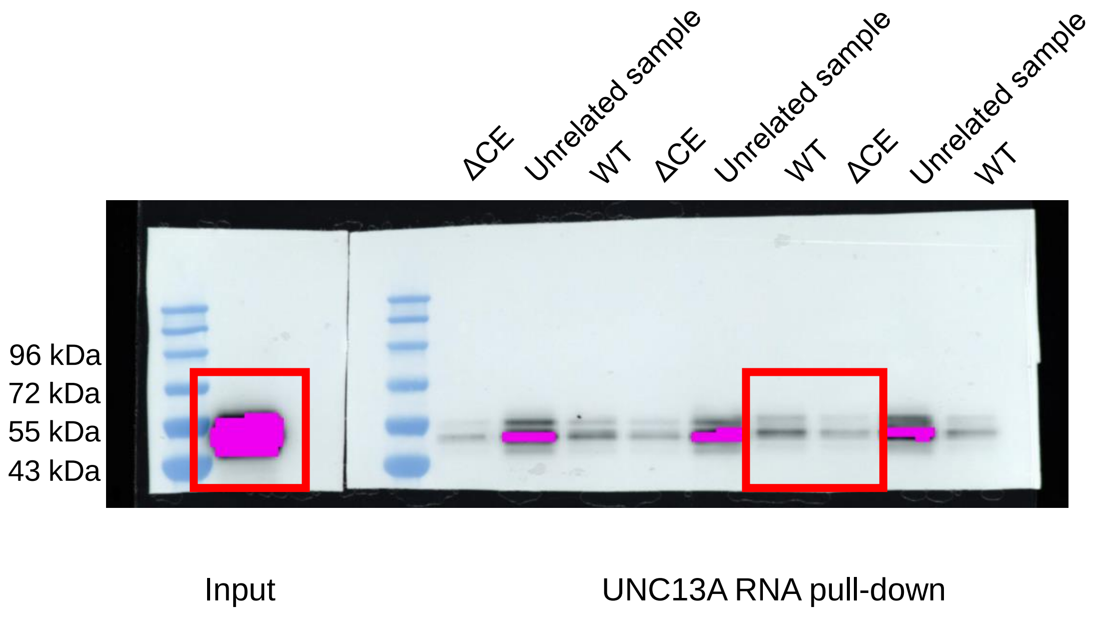

Supplement: S1 Raw images — (PDF) [file pbio.3002028.s013.pdf]
